# Predicting Protein Therapeutic Candidates for Bovine Babesiosis Using Secondary Structure Properties and Machine Learning

**DOI:** 10.3389/fgene.2021.716132

**Published:** 2021-07-23

**Authors:** Stephen J. Goodswen, Paul J. Kennedy, John T. Ellis

**Affiliations:** ^1^School of Life Sciences, University of Technology Sydney, Ultimo, NSW, Australia; ^2^School of Computer Science, Faculty of Engineering and Information Technology and the Australian Artificial Intelligence Institute, University of Technology Sydney, Ultimo, NSW, Australia

**Keywords:** *Babesia bovis*, *Babesia bigemina*, *Babesia canis*, machine learning, exportome, vaccine, protein secondary structure

## Abstract

Bovine babesiosis causes significant annual global economic loss in the beef and dairy cattle industry. It is a disease instigated from infection of red blood cells by haemoprotozoan parasites of the genus *Babesia* in the phylum Apicomplexa. Principal species are *Babesia bovis, Babesia bigemina*, and *Babesia divergens.* There is no subunit vaccine. Potential therapeutic targets against babesiosis include members of the exportome. This study investigates the novel use of protein secondary structure characteristics and machine learning algorithms to predict exportome membership probabilities. The premise of the approach is to detect characteristic differences that can help classify one protein type from another. Structural properties such as a protein’s local conformational classification states, backbone torsion angles ϕ (phi) and ψ (psi), solvent-accessible surface area, contact number, and half-sphere exposure are explored here as potential distinguishing protein characteristics. The presented methods that exploit these structural properties via machine learning are shown to have the capacity to detect exportome from non-exportome *Babesia bovis* proteins with an 86–92% accuracy (based on 10-fold cross validation and independent testing). These methods are encapsulated in freely available Linux pipelines setup for automated, high-throughput processing. Furthermore, proposed therapeutic candidates for laboratory investigation are provided for *B. bovis, B. bigemina*, and two other haemoprotozoan species, *Babesia canis*, and *Plasmodium falciparum.*

## Introduction

The underlying procedure to identify protein candidates in an *in silico* vaccine discovery pipeline is to find and exploit differences between proteins. A procedure based on a plausible assumption that proteins inducing an immune response in a host must be different to those that induce no response. More specifically, immunogenic proteins are expected to contain regions that can trigger a cellular immune response mediated by T or B cells, namely epitopes. An epitope is the minimal structure necessary to invoke an immune response and must come from proteins accessible to the immune system (Vivona et al., 2008). Several bioinformatic programs (Krogh et al., 2001; Kall et al., 2004; Horton et al., 2007; Armenteros et al., 2017, 2019a) have been developed to predict various protein characteristics given a protein’s primary structure represented by a linear sequence of amino acids. Detecting characteristic differences can help classify one protein type from another. For example, a characteristic such as whether a newly synthesised protein is targeted to the secretory pathway can be predicted from the presence of a secretory signal peptide (SP) encoded in its primary structure (Emanuelsson et al., 2007). Previous studies (Goodswen et al., 2013a,b) have collated these various predicted characteristics and then trained machine learning (ML) models to computationally detect differences, which effectively epitomises the current state-of-the-art approach to *in silico* vaccine discovery against eukaryotic pathogens.

Prediction of protein secondary structure (SS) presents further protein characterisation opportunities to help classify one protein type from another. Protein SS denotes the local conformation of a protein’s polypeptide backbone, i.e., helical and sheet hydrogen bonding patterns in a biopolymer (Yang et al., 2018). The two most common SS conformations are α-helix and β-sheet. A common SS characterisation standard (Kabsch and Sander, 1983) defines the conformation in 3 or 8 classification states according to hydrogen-bonding patterns. The 3 classes are helix, sheet, and coil commonly designated H, E, and C, respectively; and the 8 classes comprise three types for helix (G for 3_10_ helix, H for α-helix, and I for π-helix), two types for sheet (E for β-sheet and B for β-bridge), and three types for coil (T for β-turn, S for high curvature loop, and C for irregular).

Several bioinformatic programs (Jones, 1999; Magnan and Baldi, 2014; Drozdetskiy et al., 2015; Wang et al., 2016; Heffernan et al., 2018; Hanson et al., 2019; Klausen et al., 2019; Torrisi et al., 2019) have been developed to predict 3 and/or 8 classes given primary sequences. Most of these *ab initio* predictors use a combination of ML and evolutionary profiles. Variations of neural networks are the predominant ML algorithm. An evolutionary profile is derived from a multiple sequence alignment of homologous sequences (Yang et al., 2018), mainly from the position specific substitution matrix (PSSM) (Jones, 1999) calculated by Position-Specific Iterative Basic Local Alignment Search Tool (PSI-BLAST) (Altschul et al., 1997). Predictions are typically evaluated and reported as a Q3 or Q8 accuracy for 3 or 8 classes, respectively, which represents the percentage of residues correctly predicted. Although accuracies for SS predictions have steadily increased over the decades, a theoretical prediction limit of 88–90% for Q3 has been determined (Rost, 2001; Martin et al., 2005). The published predictor accuracies range from 72.5% to 87% for Q3, and 60% to 77% for Q8 (Jones, 1999; Magnan and Baldi, 2014; Drozdetskiy et al., 2015; Wang et al., 2016; Heffernan et al., 2018; Hanson et al., 2019; Klausen et al., 2019; Torrisi et al., 2019).

Other structural properties such as backbone torsion (dihedral) angles ϕ (phi) and ψ (psi), solvent-accessible surface area (ASA), contact number (CN), and half-sphere exposure (HSE) are also potential distinguishing protein characteristics. Torsion angles phi and psi provide the flexibility required for the polypeptide backbone to adopt a certain fold, and therefore determine the conformation of the backbone (Ramachandran et al., 1963). ASA provides the distinction between a buried (low ASA) and exposed (high ASA) residue to solvent (water) in its folded state (Hanson et al., 2019). CN is another solvent exposure measure that counts spatially close residues within a distance cut-off to a target residue (Pollastri et al., 2002). The distances are based on the positions of alpha carbon (Cα) or beta carbon (Cβ) atoms (Heffernan et al., 2016). HSE is a 2D measure of a residue’s solvent exposure and adds directionality to CN by splitting the spherical distance cut-off into two halves defined as upper and down (Hamelryck, 2005; Heffernan et al., 2016). Several programs (Fang et al., 2019; Hanson et al., 2019; Klausen et al., 2019) provide phi and psi angles, ASA, CN, and HSE values in their output in addition to 3 and 8 class predictions.

**FIGURE 1:**
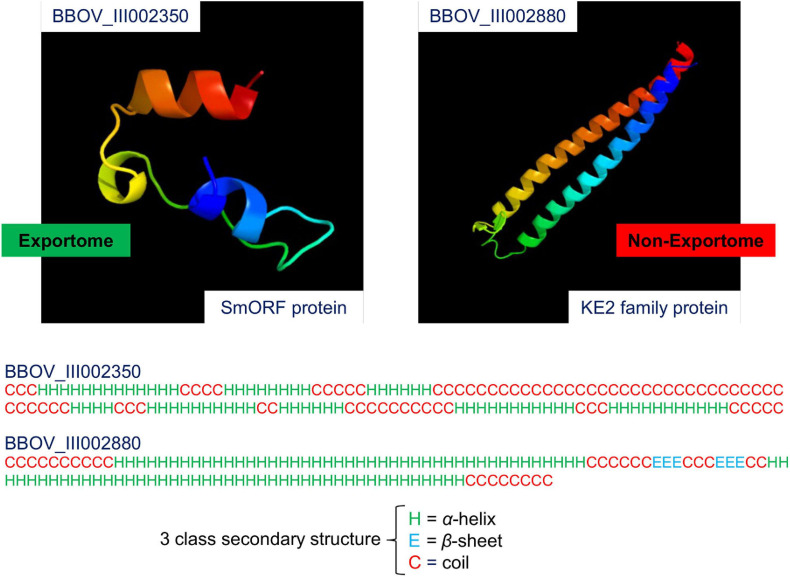
3D model of the secondary structure of two *Babesia bovis* T2Bo proteins. The two images were generated by the Phyre2 web portal for protein modelling, prediction and analysis. Protein folding is coloured by rainbow from the N to C Terminals. BBOV_III002350 is a small open reading frame (smORF) protein. It is expected to be an exportome member because smORF proteins are known to have an association with the erythrocyte membrane. BBOV_III002880 is a KE2 family protein. This protein is expected to be a non-exportome member because its subcellular location is in the cytoplasm. Note that the Phyre2 protein model reliability depends on the extent of homology between a user-supplied sequence and a sequence of known structure in the Protein Database (PDB). In this case, the BBOV_III002350 sequence has 23% coverage (33 out of 142 residues) with a known PDB molecule (nicotinate-nucleotide adenylyltransferase), and the BBOV_III002880 sequence has 83% coverage (101 out of 122 residues) with a PDB molecule (chaperone prefoldin subunit 4). Therefore, only the homologous residues are displayed in the images. Secondary-structure prediction for the three predominate states: α-helix, β-sheet, and coil are shown below the modelling images for the entire sequence lengths of BBOV_III002350 and BBOV_III002880.

*Babesia bovis* is a tick-transmitted, obligate intracellular, haemoprotozoan parasite of the phylum Apicomplexa (Homer et al., 2000). *Babesia* infection of erythrocytes (red blood cells) can cause a severe disease called babesiosis in susceptible hosts (Hunfeld et al., 2008). This disease is of interest to the current study because there is no subunit vaccine (Brayton et al., 2007) and the annual global economic loss in the beef and dairy cattle industry due to babesiosis is significant and of great concern (Suarez and Noh, 2011). Current vaccines against *B. bovis* are based on live formulations, whilst subunit vaccines are deemed safer, and easier to handle and produce (Florin-Christensen et al., 2014). Several reviews describe background and current insights into the research, detection and treatment of bovine babesiosis (Suarez and Noh, 2011; Mosqueda et al., 2012; Rathinasamy et al., 2019; Suarez et al., 2019). Potential vaccine targets against bovine babesiosis include members of the exportome, i.e., those proteins exported outside the parasite into the host’s erythrocyte cytoplasm and/or the erythrocyte membrane (Gohil et al., 2010). An unknown subset of the exportome is thought to mediate the pathogenesis of babesiosis by altering structural and functional properties of parasitised erythrocytes, and such a subset contains potential therapeutic targets (Gohil et al., 2013). Furthermore, exported proteins exposed to the immune system provide target potential for vaccine development (Rathinasamy et al., 2019). Figure 1 shows a 3D model of the SS of two *B. bovis* proteins, one expected and the other not expected to be exportome members [images generated by Phyre2 (Kelley et al., 2015)].

In a previous study, we used ML with a protein’s primary structure, principally in the input format of amino-acid composition, to predict *B. bovis* exported proteins (Goodswen et al., 2021). In this study, we investigated the novel use of protein SS characteristics to predict exportome membership as a complementary method. More specifically, trained ML models were used to detect differences in 3 and 8 state local conformations, phi and psi angles, CN, ASA, and HSE between expected exportome and non-exportome proteins. Apicomplexan pathogens, such as *B. bovis* are complex biological systems consisting of thousands of proteins. The presented ML-SS approach predicts exportome membership with 86–92% accuracy (based on 10-fold cross validation and independent testing), and therefore identifies out of thousands those proteins most worthy of further laboratory investigation. Furthermore, the approach was tested for its universal effectiveness on other parasites of the phylum Apicomplexa – three haemoprotozoan species, namely *Babesia bigemina, Babesia canis*, and *Plasmodium falciparum*. *Toxoplasma gondii*, considered the model organism for Apicomplexa (Kim and Weiss, 2004), is also tested in the current study as an outlier to *Babesia* and *Plasmodium* because it invades only nucleated cells (Sibley, 2003). Proposed candidates for laboratory investigation are provided for *B. bovis* and the three other haemoprotozoan species. Furthermore, Linux pipelines implementing the ML-SS approach are made freely available for download.

## Results

### Predicted *Babesia bovis* T2Bo Exportome Members Using Rule-Based Method

There is currently no laboratory verified list of exportome proteins for *B. bovis, B. bigemina, and B. canis.* The current state-of-the-art prediction method for exportome membership is a rule-based bioinformatics approach proposed by Gohil (Gohil et al., 2013). The rules are based on known characteristics of *Plasmodium* exportome proteins such as presence of SPs but no transmembrane (TM) domain(s) and glycosylphosphatidylinositol (GPI) anchors.

Supplementary Table 1 lists 276 out of a possible 3706 *Babesia bovis T2Bo* proteins that meet the Gohil rule-based selection criteria. Only laboratory testing can definitely confirm whether any of these 276 are exportome members. However, previous studies have provided indications of protein types likely to have an association with the erythrocyte membrane. For example, spherical body proteins (SBP) are believed to be responsible for host cell modifications (Terkawi et al., 2011; Gubbels and Duraisingh, 2012); heat shock proteins (HSP70 and HSP90) are known to be exported into the erythrocyte cytoplasm (Maier et al., 2009; Kuelzer et al., 2012); variant erythrocyte surface antigen (VESA) proteins are postulated to play a role in cytoadhesion, sequestration, and immune evasion (Allred et al., 2000; O’Connor and Allred, 2000; Brayton et al., 2007); and small open reading frame (smORF) proteins play a role in VESA protein biology (Brayton et al., 2007; Ferreri et al., 2012). The reliability of annotated protein names for *Babesia* species is considered poor by the current study (see later the section “Discussion”). Despite this, proteins are assessed here on their names. Table 1 shows a breakdown of the 276 proteins into protein types based on the annotated protein name. For example, there are four SBPs in the available 3706 *B. bovis* T2Bo proteins. Three of these fulfil the rule-based selection criteria. Two of which were previously reported to localise to the infected erythrocyte membrane (Hines et al., 1995; Ruef et al., 2000): BBOV_II002880 (SBP 1) and BBOV_I004210 (SPB 3). No SP and one TM were predicted for BBOV_II000740 (SBP 2) and consequently this protein did not meet the selection criteria. Notable findings are that most SBP, HSP70, HSP90, and smORF proteins were selected, whereas almost all VESA proteins were not due to the absence of an SP.

**TABLE 1:** Breakdown of protein types meeting the rule-based exportome selection criteria.

Protein type	Available^a^	Selected^b^
Spherical body proteins (SBP)^c^	4	3
Small open reading frame (smORF)^c^	44	42
Heat shock proteins (HSP70)^c^	2	1
Heat shock proteins (HSP90)^c^	2	2
Variant erythrocyte surface antigen-1 (VESA); family protein^c^	4	0
Variant erythrocyte surface antigen-1 (VESA); alpha subunit^c^	71	2
Variant erythrocyte surface antigen-1 (VESA); beta subunit^c^	43	0
Variant erythrocyte surface antigen-1 (VESA); putative^c^	14	1
Hypothetical proteins	1309	95
Membrane proteins (putative)	171	46
Erythrocyte membrane-associated antigen^c^	2	2
Conserved hypothetical proteins	539	20
Other	1501	62
**Total**	3706	**276**

*^*a*^Total number of available proteins of a particular protein type given 3706 *Babesia bovis* T2Bo proteins currently available.*

*^*b*^Number of proteins from available that fulfil the rule-based selection criteria for an exportome member.*

*^*c*^Protein types reported in studies to likely have an association with the erythrocyte membrane.*

Supplementary Table 1 also lists 196 proteins (∼70%) taken from the 276 fulfilling the rule-based selection criteria to represent the ‘positives’ for ML training data. The remaining 80 (∼30%) formed the independent dataset to test the ML model’s performance. Two further datasets comprising 196 and 80 proteins were selected using the Python random module from 3430 *B. bovis* T2Bo proteins that did not meet the rule-based selection criteria. These latter datasets represented the ‘negatives’ for ML training and testing, respectively.

### Predicted Exportome Members From Test Species Using Rule-Based Method

Supplementary Table 2 lists 277 out of a possible 5077 proteins currently available for *Babesia bigemina* BOND, 133 out of 3467 *Babesia canis* BcH-CHIPZ, 264 out of 5460 *Plasmodium falciparum* 3D7, and 318 out of 8322 *Toxoplasma gondii* ME49 proteins that meet the Gohil rule-based selection criteria.

Protein names of interest in those selected are ones with reported exportome associations. For example, selected *B. bigemina* proteins include HSP70, HSP90, SBP3, SBP4, and membrane attack complex (MAC)/perforin or DnaJ domain containing. Heat-shock proteins containing a DnaJ domain (previously known as HSP40s) are reported to export into the erythrocyte cytoplasm and be involved in transport of parasite proteins, including *P. falciparum* erythrocyte membrane protein 1 (PfEMP1) (Maier et al., 2009). Apicomplexan MAC/perforin proteins have been shown to be secreted from micronemes during the intraerythrocytic parasite stage and bind to the parasitised erythrocyte membrane, where it facilitates the egress of the parasite (Paoletta et al., 2021).

Selected *B. canis* proteins include HSP90 and MAC/perforin. Selected *P. falciparum* proteins include HSP70, HSP90, HSP110, HSP20-like chaperone, and *Plasmodium* exported proteins containing helical interspersed subtelomeric (PHIST) or HYP domains. Proteins containing PHIST and HYP domains are exported to the infected erythrocyte membrane (Oberli et al., 2014; Schulze et al., 2015).

A notable protein type not selected was PfEMP1 because of the absence of SPs. PfEMP1 is known to play a role in erythrocyte modification (Cooke et al., 2006). Selected proteins containing PHIST domains are known, however, to bind to PfEMP1 (Oberli et al., 2014). Other selection exceptions are proteins from two *Plasmodium* protein families, repetitive interspersed family (RIFIN) and subtelomeric variable open reading frame family (STEVOR), which are thought to play roles in export and display of virulence proteins (Maier et al., 2009; Haase and de Koning-Ward, 2010). Although most RIFIN and STEVOR proteins have SPs, all but one STEVOR-like protein fails the selection criteria due to the presence of at least one TM.

Selected *T. gondii* proteins include dense granule (GRA) and rhoptry (ROP) proteins, which are categorised as excreted/secreted proteins and not exportome members as *T. gondii* parasites do not live in erythrocytes. GRAs and ROPs are known to be excreted/secreted into the parasitophorous vacuole and/or host cell from their respective subcellular organelles, rhoptries and dense granules (Hakimi et al., 2017). Only 12 out of 37 GRA and ROP proteins meet the selection criteria.

### Predictors for 3 and 8 Classes

Nine 3 class and seven 8 class conformational state predictors were used in this study. The output from each predictor shows at least 3 and/or 8 structural classifications for every amino acid in the primary input sequence, e.g., each amino acid is classified H, E, or C for 3 classes and G, H, I, E, B, T, S, or C for 8 classes. The predictors were evaluated by comparing a consensus from all predictors with the individual predictor’s classifications given the 392 training data protein sequences as input (i.e., 196 positives + 196 negatives). Table 2 shows the percentage of classifications for each predictor that matched the consensus classification. For example, 94.0% of Porter 5 classifications for 3 class predictions matched the consensus classifications derived from all nine predictors. The true secondary structures of the training proteins are unknown and consequently the consensus accuracies are unknown. The percentages in Table 2 are therefore not a true indication of a predictor’s accuracy. However, the assumption here is that predictors making the most similar predictions are more accurate than outlier predictors. With this assumption, Porter 5 is the most and DeepCNF is the least accurate for both 3 and 8 class predictions.

**TABLE 2:** Percentage of secondary structure classifications matching a consensus classification.

Predictor	Class 3 (%)	Class 8 (%)
DeepCNF	77.4	70.8
Spider3^a^	79.7	71.7
Jpred 4^b^	83.8	
SSpro	86.3	77.0
NetsurfP	87.1	82.1
PSIPRED^b^	90.2	
MUFold	91.2	87.2
SPOT-1D	92.0	88.5
Porter 5	94.0	90.4

*Class 3, predictors that predict three class states – helix, sheet, and coil; Class 8, predictors that predict eight class states – three types for helix (3_10_ helix, α-helix, and π-helix), two types for sheet (β-sheet and β-bridge), and three types for coil (β-turn, high curvature loop, and irregular).*

*^*a*^Single-sequence-based prediction version (i.e., uses no multiple sequence alignment of homologous sequences).*

*^*b*^These programs do not predict 8 Class secondary structure states.*

### Predicted Exportome Members Using Machine Learning With 3 and 8 Class Predictions

**FIGURE 2:**
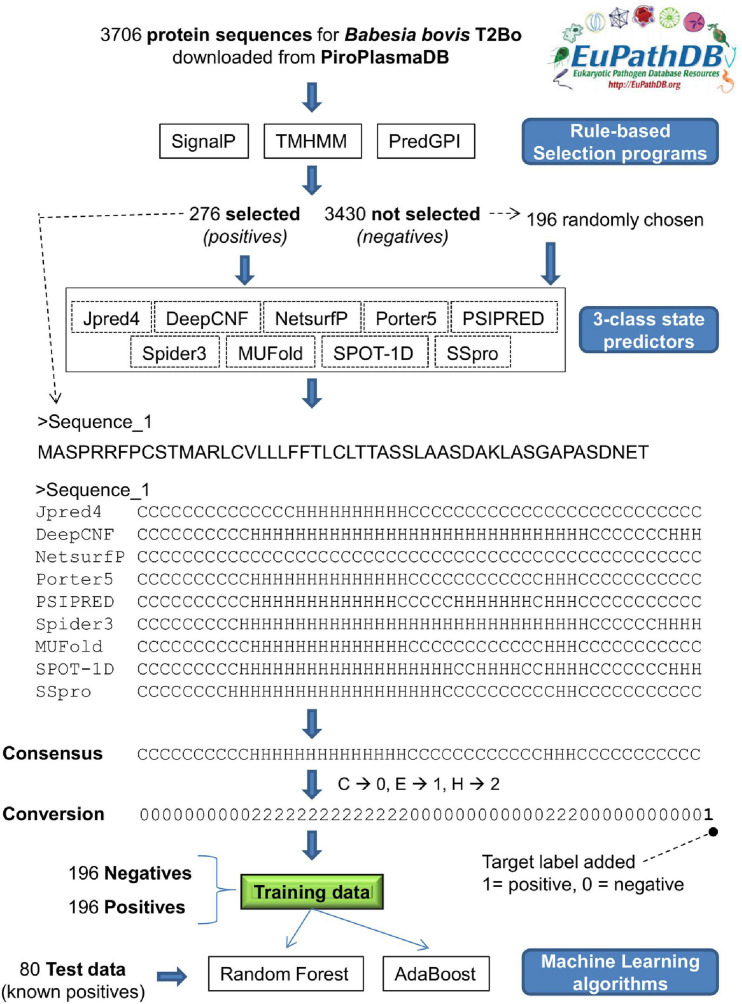
Schematic of steps taken to determine the exportome membership of *Babesia bovis* T2Bo proteins using machine learning and three-class state predictions for protein secondary structure. (1) Following a rule-based selection procedure, protein sequences from 276 proteins fulfilling the selection criteria (positives) + 196 failing the selection criteria (negatives) are input to nine 3-class conformational state predictors, (2) a consensus of the nine individual predictions is determined; (3) consensus characters are converted to numeric values in preparation for machine learning input, (4) a ‘1’ or a ‘0’ is appended to the numerical consensus as an indication it represents positive or negative training data, respectively; (5) a training dataset is collated comprising 196 positives and 196 negatives; (6) machine learning algorithms, Random Forest and Adboost, are trained using the training dataset; (7) the exportome membership of 80 positives (276 – 196) is predicted using the trained machine learning models.

Figure 2 shows the steps taken to classify 392 *B. bovis* T2Bo proteins as either an exportome (positive) or non-exportome (negative) using ML and protein SS classifications. Protein sequences from 392 proteins, which represent the training data, were input into nine 3 class and seven 8 class predictors. Consensus classifications were derived from the predicted 3 and 8 classifications from each predictor. These consensus classifications were subsequently used to train the ML algorithms, adaptive boosting (adaBoost) and Random Forest (RF). Different representations of data input to the ML algorithms were evaluated using 10-fold cross validation (Materials and methods describes each representation). The best performances were derived when using only the first 40 classifications from the N-terminal and a proportional class count for the remaining classifications. Table 3 shows the ML performance measures obtained from 10-fold cross validation. For example, an ensemble of ML algorithms consisting of adaBoost and RF given 3 and 8 class consensus predictions achieved 86.99% and 86.73% accuracies, respectively, in classifying 392 *B. bovis* proteins as either a positive or negative. Supplementary Data 1 shows the ML performances for all the data input representations.

**TABLE 3:** Machine learning performance measures for predicting exportome membership using 3 and 8 class conformational state predictions.

	Class 3			Class 8		
Performance measures (%)	adaBoost	RF	Ensemble	adaBoost	RF	Ensemble
Accuracy	86.73	87.24	86.99	86.48	85.71	86.73
Error rate	13.27	12.76	13.01	13.52	14.29	13.27
Sensitivity	88.27	89.29	87.76	88.27	87.24	87.76
False positive rate	14.80	14.80	13.78	15.31	15.82	14.29
Specificity	85.20	85.20	86.22	84.69	84.18	85.71
Positive predictive value	85.64	85.78	86.43	85.22	84.65	86.00
Negative predictive value	87.89	88.83	87.56	87.83	86.84	87.50

*adaBoost, adaptive boosting; RF, Random Forest; Ensemble, final classifications derived from the average of adaBoost and RF classification probabilities; Class 3, predictions based on three class states; Class 8, predictions based on eight class states.*

### Predicted Exportome Members Using Machine Learning With Phi and Psi Angles

Torsion angles phi and psi determine protein conformation, which in turn determines the protein function (Fang et al., 2019). The premise here is that a different conformation exists between exportome and non-exportome proteins, and therefore a difference in psi and phi angles. A ‘Psi vs. Phi’ angle plot is shown in Supplementary Figure 1. This plot is similar to a Ramachandran plot (Ramachandran et al., 1963).

Three predictors (SPOT-1D, NetsurfP, and MUFold) were used to predict psi and phi angles for the 392 *B. bovis* T2Bo training proteins. Outputs from the predictors are values between −180 and +180 for each amino acid in the input sequence and represent the psi and phi angles of the protein. The mean psi and phi angles at each amino acid were determined using the values from each predictor. As a predictor comparison measure, the absolute difference between the mean angle and the predictor’s predicted angle was determined at each amino acid and summed. MUFold had the least total deviation from the mean for psi angles, followed by SPOT-1D, then NetsurfP; and SPOT-1D had the least total deviation for phi angles, followed by NetsurfP, then MUFold.

There was no detectable difference observed in the mean angles between the 196 exportome and 196 non-exportome proteins and hence the application here of ML. Various representations of the two sets of angles (psi and phi) as a uniform set of features for ML input were assessed with 10-fold cross validation (Materials and methods describes each representation). The representation that achieved the best accuracy of 86.73% was obtained by using the psi and phi angles from the first 40 amino acids (AAs) to in effect have 80 features per protein. Table 4 shows the ML performance measures obtained from 10-fold cross validation for this representation when classifying the 392 training proteins as either positives or negatives. Supplementary Data 1 shows the ML performances for all the data input representations.

**TABLE 4:** Machine learning performance measures for predicting exportome membership using backbone torsion angles ϕ (phi) and ψ (psi), half-sphere exposure (HSE) upper sphere predictions, and solvent-accessible surface area (ASA).

	ϕ (phi) and ψ (psi)			HSE_u			ASA		
Performance measures (%)	ada	RF	Ens	ada	RF	Ens	ada	RF	Ens
Accuracy	86.73	84.85	86.73	89.54	91.84	90.31	90.31	88.78	90.31
Error rate	13.27	15.05	13.27	10.46	8.16	9.69	9.69	11.22	9.69
Sensitivity	86.73	80.10	86.22	92.86	93.37	93.37	93.37	90.82	93.37
False positive rate	13.27	10.20	12.76	13.78	9.69	12.76	12.76	13.27	12.76
Specificity	86.73	89.80	87.24	86.22	90.31	87.24	87.24	86.73	87.24
Positive predictive value	86.73	88.70	87.11	87.08	90.59	87.98	87.98	87.25	87.98
Negative predictive value	86.73	81.86	86.36	92.35	93.16	92.93	92.93	90.43	92.93

*ϕ (phi) and ψ (psi), backbone torsion (dihedral) angles; HSE_u, half-sphere exposure (HSE) upper sphere; ASA, solvent-accessible surface area; ada, adaptive boosting; RF, Random Forest; Ens, ensemble = final classifications derived from the average of adaBoost and RF classification probabilities.*

### Predicted Exportome Members Using Machine Learning With the Secondary Structure Properties ASA, CN, and HSE

Predicted SS properties of ASA, CN, and HSE (for both upper and down spheres) were used in turn as ML input features to classify the 392 *B. bovis* T2Bo proteins. ASA and HSE-upper predictions from the first 40 AAs achieved the best ML performance measures as determined by 10-fold cross validation with an equal accuracy of 90.31% (see Table 4). HSE-down features obtain the next best accuracy followed by CN (shown in Supplementary Data 1).

### Comparison of Secondary Structure Derived Machine Learning Methods

The exportome membership probabilities predicted by each of the best performing ML SS prediction methods during 10-fold cross validation and testing are shown in Supplementary Table 3. The five best performing methods comprise 3 and 8 classes, psi and phi angles, ASA, and HSE-upper – referred to henceforth as the ML-SS methods. Each protein was reclassified from that expected based on a 0.5 probability threshold for comparative purposes. The percentage of classifications per method different to that expected is shown in Table 5. For example, the least percentage of positive misclassifications observed during cross validation was 6.1% for both ASA and HSE-upper predictions, and the least percentage of negative misclassifications was 12.8% for ASA, HSE-upper, and psi and phi angles predictions. The percentage of misclassifications reduces to 4.6% and 2.0% for positives and negatives, respectively, when classifications are based on the average of the exportome membership probabilities from all five methods. Using the ‘average’ effectively increases the prediction accuracy to 96.7%. A misclassification consensus was also determined, e.g., ‘0’ indicated all five methods classified a protein as expected, and ‘5’ indicated all five methods misclassified a protein to that expected (see Supplementary Table 3). One positive protein (BBOV_IV011310 – membrane protein; putative) was misclassified by all five methods and; 71.4% and 62.5% of positives and negatives, respectively, were classified as expected by all five methods.

**TABLE 5:** Percentage of prediction misclassifications and the accuracy per secondary structure prediction method for *Babesia bovis* T2Bo training and test data.

	Training data ^b^			Test data ^c^		
Prediction method	Positives (%)	Negatives (%)	Accuracy (%)	Positives (%)	Negatives (%)	Accuracy (%)
3 classes	12.2	14.1	87.0	10.0	11.3	89.4
8 classes	12.2	14.2	86.7	8.8	15.0	86.3
Phi and psi angles	13.8	12.8	86.7	13.8	11.3	87.5
Solvent-accessible surface area	6.1	12.8	90.3	3.8	10.0	93.1
Half-sphere exposure – upper sphere	6.1	12.8	90.3	2.5	12.5	92.5
**Average ^a^**	**4.6**	**2.0**	**96.7**	**3.8**	**5.0**	**95.6**

*^*a*^Average = average exportome probabilities for all five prediction methods (3 classes, 8 classes, phi and psi angles, solvent-accessible surface area, and half-sphere exposure – upper sphere).*

*^*b*^Training data = 196 *Babesia bovis T2Bo* proteins that meet the Gohil rule-based selection criteria (positives), and 196 *Babesia bovis T2Bo* proteins that fail the Gohil rule-based selection criteria (negatives).*

*^*c*^Test data = 80 *Babesia bovis T2Bo* proteins that meet the Gohil rule-based selection but not included in training data (positives), and 80 *Babesia bovis T2Bo* proteins that fail the Gohil rule-based selection criteria but not included in training data (negatives).*

*Positives (%) = percentage of positive classifications per method different to that expected; Negatives (%) = percentage of negative classifications per method different to that expected.*

Table 5 also shows the percentage of misclassifications per prediction method when using *B. bovis* T2Bo test data as input to the five methods. Test data consisted of 80 positive and 80 negative proteins not used in training. All misclassification percentages were expected to slightly decrease due to using the full training data and this expectation was observed. Similarly, the accuracies for each method increased as expected. Using the ‘average’ to represent all five prediction methods reduced the percentage of misclassifications and improved the overall accuracy to 95.6%. No proteins were misclassified to that expected by all five methods, but one positive (BBOV_IV000520 – importin beta subunit, putative) and three negative proteins BBOV_II005340 – cytidine triphosphate synthetase, putative; BBOV_III005850 – membrane protein, putative; and BBOV_III003780 – conserved hypothetical protein) were misclassified by four methods. The highest scoring positive was a smORF protein when based on the average score, whereas the lowest scoring positives have protein names not known to be associated with the erythrocyte membrane.

As a further evaluation to verify that the results did not occur by chance but were attributable to input values at specific locations, the input values to the ML-SS methods were randomly shuffled. The prediction accuracies based on random shuffling obtained from 10-fold cross validation were 56.1%, 57.9%, 54.9%, 73.2%, and 71.7% for 3 class, 8 class, psi and phi angles, ASA, and HSE-upper, respectively.

**FIGURE 3:**
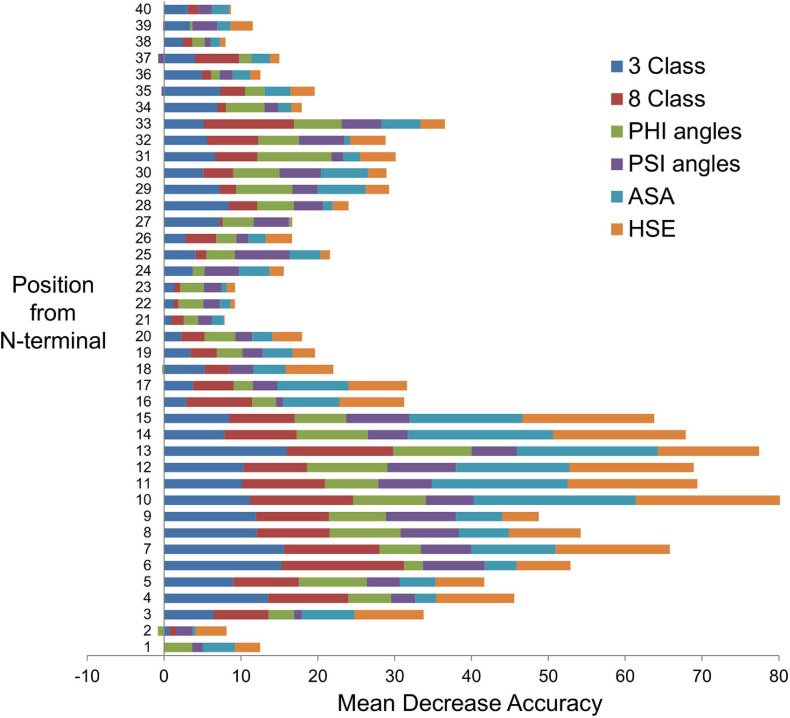
Feature importance from secondary structure prediction methods. The bar chart shows the feature importance from each of the secondary structure prediction methods: 3 Class, three local conformational states; 8 Class, eight local conformational states; PHI angles, torsional (dihedral) angle ϕ (phi); PSI angles, torsional (dihedral) angle ψ (psi); ASA, solvent-accessible surface area; HSE, half-sphere exposure (upper). Position from N-terminal is equivalent to the amino acid number in a protein sequence. Each position is a machine learning input feature. There are 40 features in this example. The Random Forest algorithm has built-in feature importance function that computes ‘mean decrease accuracy’, which is based on how much the accuracy decreases when the feature is excluded, i.e., a measure of the feature contribution toward the Random Forest model’s prediction accuracy – the greater the contribution, the higher the importance.

Figure 3 shows a comparison of each feature contribution toward the ML-SS methods’ prediction accuracies. For example, SS prediction values for the first 40 AAs from the N-terminal represent 40 input features to the ML-SS methods. Features in positions 10–15 have the most predictive importance. The predicted SP cleavage sites for the 276 expected exportome members range from 13 to 37 AAs measured from the N-terminal (average 22.5 AAs). Feature at position 21 makes the least contribution, with the greatest contributions from features in the SP regions. Interestingly however, if ML input features are restricted to only the first 25, the prediction accuracies reduce to 84.7%, 85.7%, 85.3%, 89.0%, and 88.8% for 3 class, 8 class, psi and phi angles, ASA, and HSE-upper, respectively. This suggests that regions beyond the SP cleavage sites contain additional, albeit weaker signals that contribute to differentiating between positives and negatives, especially positions 28–33.

### Predicting the Presence of Transmembrane Domains Using Machine Learning and Secondary Structure Characteristics

All five SS predictions methods revealed during the 10-fold cross validation and testing that the first 40 AAs encodes the strongest signal for differentiating between positives and negatives. Most TMs are not located in the first 40 AAs. The presence or absence of a TM therefore provided little or no contributing signal to the overall prediction outcomes. The expectation still remains that a protein with one or more predicted TMs is a less worthy exportome candidate than a protein with no TMs. We therefore investigated the use of SS predictions and ML to specifically predict the presence of TMs. Supplementary Table 4 lists all *B. bovis* proteins containing at least one TM as predicted by TMHMM, and a TM presence probability. This ML-derived probability was obtained by counting the number of AAs per protein that fall into a particular location on ‘Psi vs. Phi’ angle plot, although other variations of fixed sets of values for ML input were evaluated (see the section “Materials and Methods”). The training data was collated from predictions determined by TMHMM because of the limited experimental evidence for TMs in *B. bovis* proteins. Using predicted TMs may be deemed counterintuitive, but it served the investigative purpose of determining whether the presence or absence of TMs could be represented by SS predictions. Supplementary Table 5 shows the ML performance measures. The best achieved accuracy was 76.25%. It is concluded that the ‘Psi vs. Phi’ ML-derived probabilities could be used to complement the ML-SS methods by providing a TM presence indicator.

### Predicted Exportome Members for All *Babesia bovis* T2Bo Proteins Using Machine Learning Methods

All 3706 *B. bovis* T2Bo proteins minus the 392 training proteins (equating to 3314) were input into the ML-SS methods. Only Spider3 (Heffernan et al., 2018) was used to generate the required SS prediction inputs (3 and 8 classes, psi and phi angles, ASA, and HSE-upper). Spider3 is a single-sequence-based prediction method that does not required evolutionary information from multiple sequence alignments. The 3314 input included the 80 positive and 80 negative test proteins. Supplementary Table 6 shows the percentage of prediction misclassifications when using Spider3 only as a comparison to multiple predictor inputs (as per Table 5). The source of the 3 and 8 classes, and the psi and phi angle predictions underlying Table 5 were obtained from a consensus of multiple predictors; but ASA and HSE-upper predictions were also from Spider3. In summary, the accuracy per method for the test proteins when using Spider3 inputs was 82.5% (3 classes), 83.1% (8 classes, and psi and phi angles), 91.9% (HSE-upper), and 93.1% (ASA). All these accuracies are lower than those shown in Table 5. Despite lower accuracies for each method when using only Spider inputs, the overall accuracy was comparable with 96.2% when using the ‘average’ to represent all five prediction methods. These accuracies were computed with a 0.5 threshold. We propose more stringent selection criteria when using the presented ML-SS methods for predicting therapeutic candidates for laboratory investigation: an exportome membership probability greater or equal to 0.7, a TM presence indicator less than 0.5 (i.e., no predicted TM), and a consensus of four or more SS prediction methods. Such a selection criteria applied to the 160 test proteins would incorrectly filter 25% positives, and incorrectly include 1.3% negatives. However, less false positives in the laboratory would be expected.

Supplementary Table 3 shows the predicted exportome probabilities for each of the 3314 *B. bovis* proteins. Out of 3154 proteins (i.e., 3314 – 160 testing proteins = 3154), 101 candidates were selected based on the proposed stringent criteria. Supplementary Table 7 shows a breakdown of the 3314 selection pool in terms of predicted SPs and TMs. Out of the selected 101 candidates, 75 have no SPs and TMs. Also, 19 of the 101 (18.8%) contain more than 1 TM as predicted by TMHMM, which are considered here as possible false positives. Most of the candidate protein names are hypothetical, putative or are not known to be associated with erythrocyte membranes. However, one candidate exception is a smORF protein.

Supplementary Table 3 also lists, as a comparison, the predicted exportome membership probabilities derived from both the ML-SS methods and an alternative independent method. This alternative method uses ML with input comprising amino acid composition and delivery signals as described in a previous study (Goodswen et al., 2021). Out of 3109 *B. bovis* proteins, 85.37% have the same exportome or non-exportome predicted outcome based on a 0.5 threshold (the comparison excluded all proteins used in training). When using an average of both methods’ probabilities, 71 proteins have an average greater or equal to 0.7. A smORF and a SBP2 are the only high average probability proteins with known exportome names.

### Predicted Exportome Members From Test Species Using Machine Learning and Secondary Structure Properties

Supplementary Table 8 lists predicted exportome membership probabilities of all proteins that meet the Gohil rule-based selection criteria from the study’s four Apicomplexa test species. These probabilities were predicted by the ML-SS methods with the *B. bovis* T2Bo training data (i.e., 196 positives + 196 negatives). Supplementary Table 9 compares summarised probability counts from the four test species. Based on an average exportome membership probability greater than 0.5, 92.1% of the *B. bigemina* BOND, 87.9% of *B. canis* BcH-CHIPZ, 98.1% of *P. falciparum* 3D7 rule-based derived exportome proteins are predicted exportome members by the ML-SS methods. The results suggest that patterns presented within the SS properties that define expected exportome and non-exportome proteins in *B. bovis* are universal to the test species. For the outlier species *T. gondii*, 86.5% of the rule-based selected proteins had a probability greater than 0.5.

*Plasmodium* RIFIN and STEVOR proteins are of interest because of their association in export and display of virulence proteins. No RIFINs and only one STEVOR protein fulfils the rule-based selection criteria. Interestingly, the ML-SS methods predicted 155 out of 157 RIFINs (52 with no SP) and 30 out of 33 STEVORs (11 with no SP) to be exportome members.

Supplementary Table 8 also lists exportome membership probabilities for every currently available protein from the four test species. These probabilities were predicted by the ML-SS methods with the *B. bovis* T2Bo training data and Spider3 input. Supplementary Table 10 summarises the number of proteins per species proposed as candidates worthy of further investigation. The numbers presented are governed by the thresholds applied to both exportome membership and TM presence probabilities. For example, there is a greater number of proposed candidates but with potentially more false positives than negatives when using lower thresholds. With the stringent selection criteria previously defined, we propose 327 *B. bigemina*, 155 *B. canis*, and 372 *P. falciparum* candidates for further investigation (see Supplementary Table 8). These candidates consist of 39.0% with no SP as predicted by SignalP and 15.6% with a TM as predicted by TMHMM.

## Discussion

A subunit vaccine is urgently required to alleviate the significant annual global economic loss in the beef and dairy cattle industry due to babesiosis. The foremost objective of the current study was to identify using an *in silico* approach the most worthy therapeutic candidates from potentially thousands of *B. bovis* proteins. More specifically, identify candidate members of the exportome, which are expected therapeutic targets against babesiosis. Candidate proteins accessible to the immune system are potential subunit vaccines. The identified candidates provide an important starting impetus for downstream laboratory investigations. To appropriately place this objective into perspective, it needs to be emphasised that *B. bovis* is a complex biological system with a multifaceted life cycle that infects an even more complex biological system in the form of cattle. The reality of such an infection is the interaction of a multitude of specialised molecules in a three dimensional (3D) environment. Our objective essentially attempts to predict from a digital linear abstraction of a protein molecule, represented as a sequence of letters, whether it will induce memory helper T and B cells when incorporated in a vaccine formulation.

The current state-of-the-art approach to *in silico* vaccine discovery against eukaryotic pathogens is to use trained ML models to detect differences between predicted protein characteristics representing candidates (positives) and non-candidates (negatives). This study investigated whether differences in predicted SS characteristics between proteins representing exportome and non-exportome members could be detected with ML. Using SS characteristics for this purpose is a novel approach that is considered complementary to the current one. SS properties such as α-helixes, β-sheets, torsion angles phi and psi, ASA, CN, and HSE are related to the 3D structure of a protein. A protein’s 3D structure determines its function and accessibility to the immune system. The premise of the presented approach is that exportome and non-exportome members have different 3D structures and these differences may be detected in their underlying SS properties.

Our pathogen of interest is *B. bovis* for which there is no subunit vaccine. Furthermore, there is currently no laboratory verified list of exportome proteins for *B. bovis* or even for closely related species *B. bigemina* and *B. canis*. This presented an unavoidable challenge to the study in that there are no verified data for ML training or validating the predictions. One solution considered was to use protein types shown in previous studies to have an association with the erythrocyte membrane, i.e., use proteins ‘expected’ to be exportome members. However, the number of these expected proteins reported in published studies is limited and provides an insufficient number for ML training. The cyclic conundrum is that a sufficient number of verified target candidates are required to predict target candidates. It therefore should be acknowledged that our approach, as is the initial case with all *in silico* vaccine discovery approaches, requires iterative cycles of ML predictions, laboratory feedback and training data adjustment. Our study provides the ML predictions to help initiate this required cyclic approach.

Predicting ‘expected’ exportome proteins using extant bioinformatic programs was deemed the best solution for obtaining the initiating training data. Expected proteins are those exported outside the parasite. The presence of SPs and the absence of TMs and GPI-anchors provide indications that a protein may be secreted beyond the parasite membrane. In this study, the programs SignalP, TMHMM, and PredGPI predicted SPs, TMs, and GPI-anchors, respectively. A rule-based approach applied to these program outputs determined the positive and negative training data classification. We acknowledge the following limitations of our approach to obtaining this training data: (1) unknown misclassifications owing to the inherent imprecise nature of all prediction programs; (2) the limitation of rule-based systems *per se* because they tend to fail outside test scenarios with unseen data; (3) the ambiguity of the SP rule when the presence of an SP is only an indication a protein is targeted to the secretory pathway and not necessarily beyond the parasite membrane, and SP-containing proteins are known to be of two distinct types: those secreted from organelles during erythrocyte invasion, and those exported and involved in erythrocyte modification; and (4) the approach excludes proteins without SPs that are exported by distinct non-classical secretion pathways, although non-classical pathway *Babesia* proteins associated with erythrocyte membranes are yet to be reported.

The limitations of the rule-based approach further exacerbated the challenge of validating the predictions derived from the ML-SS methods. That is, there was an inept unavoidable scenario where unverified rule-based predictions were used not only for ML training but validation. Conversely, these rule-based limitations in the current approach for predicting exportome membership instigated our motivation for using ML with SS properties as an alternative approach. Our premise was that the feasibility of using SS properties could still be evaluated despite an unknown percentage of misclassified training data because some ML algorithms have the capacity to detect informative classification signals despite noisy or inconsistent data.

Nine SS predictors were evaluated and used in varying degrees in this study. As highlighted in Table 2, SS predictions varied for each predictor from 6% to 22.6% from a consensus prediction, given the training data sequences as input. These prediction variations are supported by the published predictor accuracies that range from 72.5% to 87% for Q3, and 60% to 77% for Q8. These inaccuracies *per se* add to an increasing accumulation of inaccuracy commencing from the genome sequencing to the translation of predicted genes to protein sequences. Our premise, however, is that the same level of SS prediction inaccuracies exist in both negatives and positives, irrespective of magnitude. In other words, the role of the SS predictions here is to represent structural patterns for differentiating between protein types and not for an accurate study of a protein’s true conformation, e.g., SS predictions such as for phi and psi angles, ASA, CN, and HSE are continuous numerical values. The importance to this study is how adequately these values of a particular predictor represent structural patterns, regardless whether a value itself is more or less accurate than another predictor’s value.

The training data protein sequences were input into various SS property predictors. Predictions for seven types of properties (3 and 8 classes, phi and psi angles, ASA, CN, and HSE down and upper spheres) were formatted into one file per property and evaluated separately. An appropriate format for ML input requires a uniform set of features. *Babesia bovis* proteins vary in length from 38 to 4820 AAs. This necessitated either fixed length inputs from each protein or property counts for the entire protein to fulfil the ML input format requirement. Variations of fixed sets of features comprising different representations of the seven property types were evaluated using 10-fold cross validation. The best performances on the independent test dataset in terms of accuracy per property type were, in ascending order: CN (83.7%), HSE down sphere (83.9%), 8 classes (86.3%), phi and psi angles (87.5%), 3 classes (89.4%), HSE upper sphere (92.5%), and ASA (92.5%). The prediction accuracy increases to 95.6% when classifications are based on the average of the exportome membership probabilities from the best five property types.

The ML input representation achieving the best performance for each property type was when using the first 40 property values from the N-terminal with a proportional count of values for the remaining protein length. This finding elicits two important issues. SPs are mainly located in the first 60 AAs, and most TMs are not located in the first 40 AAs. This implies that the ML algorithms are differentiating between positives and negatives based purely on the presence or absence of SPs. An implication that is not unexpected because all the positive training data comprises proteins with SPs. In fact, of the 368 *B. bovis* proteins predicted to contain an SP irrespective of TMs, 276 are used in the training and test data. This presents a challenge in determining whether there is an encoded signal specific to exportome proteins in addition to SPs. A proposal for future research when many more verified exportome proteins are known is to use an equal proportion of SP-containing proteins in both positives and negatives. For instance, positives would consist of known exportome proteins, where a proportion is expected to have SPs; and negatives would be non-exportome proteins but with an equal proportion containing SPs, e.g., those SP-containing proteins that invade erythrocytes. Given ML training data with a proportional number of SPs, a specific exportome signal should be unambiguously detectable, if one existed.

Exportome membership probabilities were predicted using the ML-SS methods for every available *B. bovis* protein minus those used for training and testing. From these predictions, 101 candidates were selected on stringent criteria as the most worthy for further investigation. Interestingly, 75 out of the 101 do not have a predicted SP, which included a SmORF protein. It is possible these 75 proteins were incorrectly predicted to have no SP by SignalP, but equally possible to possess similar SS properties to those in true exportome proteins. Furthermore, 26 out of 101 candidates have a TM. Likewise, these TMs may be incorrectly predicted by TMHMM and the ML algorithms have correctly detected SS patterns similar to those presented by exportome proteins.

A further challenge to the study was the uncertainty in the *B. bovis* annotation quality of protein names (see Annotation analysis in section “Materials and Methods”). The poor annotation had two implications. First, protein names appeared to contradict expectations following the selection of the training data based on the rule-based approach. For example, VESA named proteins were in both negative and positive datasets. Second, appraising the prediction methods based on protein names is potentially inaccurate given the poor annotation. Consequently, protein sequences took precedence over names in this study, despite sequences having their own levels of inaccuracies. The high scoring exportome proteins presented in the results must therefore come with a caveat that their names may be misleading with regard to their sequence signals encoded and true function.

Most of the SS predictors use PSI-BLAST to create an evolutionary profile. PSI-BLAST can take about 30 minutes (especially for SPOT1D) to process a short protein around 100 AAs and up to multiple hours for sequences greater than 1000 AAs (performed on a HPC computer with 64 bit kernel, 32 MB memory, and 8 cores). The average *B. bovis* protein length is 500 AAs. This makes the desired high-throughput processing a considerable drawback unless lengthy computational times are not an issue. Our proposal is to use only Spider3 predictions, which does not use PSI-BLAST and can process thousands of proteins in minutes. Spider3 predictions (especially 3 and 8 classes, and psi and phi angles) were observed to be less accurate in comparison to the other eight SS predictors. Nonetheless, our premise that the same level of SS prediction inaccuracies exist in both negatives and positives appears to be upheld, i.e., an appropriate level of SS pattern differences were detectable as supported by a binary classification accuracy of 96.2% when using the test dataset.

The ML-SS methods with *B. bovis* T2Bo training data were used to predict exportome membership probabilities for every available protein from four Apicomplexa test species. Classification accuracies between 86.5 and 92.1% on proteins fulfilling the Gohil rule-based selection criteria suggested that patterns presented within SS properties defining *B. bovis* expected exportome and non-exportome members are universal to the test species. Furthermore, the large percentage (39.0%) of proteins with exportome membership probabilities >0.7 and no SP supports the possibility there is a SS pattern specific to exportome proteins in addition to a SS pattern representing SPs.

We conclude that representing the ‘secondary’ structure of proteins as a set of features for ML algorithms, in particular RF and adaBoost, provides the potential to classify apicomplexan exported from non-exported proteins with an accuracy of 86–92% accuracy (based on 10-fold cross validation and an independent test dataset). It is problematic, however, to decisively claim here that the presented ML-SS methods are superior to the rule-based approach due to the rule-based origins of the training data. The lack of verified *B. bovis* exportome proteins for training and testing posed the study’s main challenge. Paradoxically, the lack of known candidates and the urgency for a *B. bovis* vaccine provided the motivation for the study. At least three *B. bovis* proteins (BBOV_II007340, BBOV_II002880, and BBOV_I004210) have been experimentally verified in a previous study (Gohil et al., 2013) to be associated with the infected erythrocyte membrane. All three proteins were predicted by the ML-SS methods to have exportome membership probabilities greater than 0.7. This further supports, albeit with a small sample, the potential of SS representations and ML.

The current study focused on proteins from the exportome of *B. bovis* with the aim of identifying therapeutic candidates. We acknowledge, however, there are other protein types that are potential candidates, e.g., proteins involved in the invasion of erythrocytes. Proteins of this type are accessible to the immune system and several representatives of the *Babesia* species have been shown to induce an immune response, namely merozoite surface antigen-1 (MSA-1) (Elisa Rodriguez et al., 2014), thrombospondin-related anonymous protein (TRAP) (Gaffar et al., 2004; Gonzalez et al., 2019), rhoptry associated protein 1 (RAP-1) (Norimine et al., 2003; Gonzalez et al., 2019), and Erythrocyte binding protein (Abd El-Salam El-Sayed et al., 2017).

We make available via GitHub, five independent pipelines that use five different predicted SS properties to predict exportome membership. The five properties are: 3- and 8-state SS predictions, phi and psi angles, and HSE-upper. Furthermore, exportome membership probabilities are provided for every available *B. bovis* T2Bo, *B. bigemina* BOND, *B. canis* BcH-CHIPZ, and *P. falciparum* 3D7 proteins. The expectation is that a desired percentage of high probability candidates can be selected to suit laboratory capability and budget. These candidates help initiate the required iterative cycles of laboratory testing, training data adjustment (i.e., adding or removing verified proteins), and further ML-SS predictions.

## Materials and Methods

### Data Source

All 3706 and 5077 currently available protein sequences for *B. bovis* T2Bo and *B. bigemina* BOND, respectively, were downloaded in a FASTA format from PiroPlasmaDB (release 47), which is a database member of Eukaryotic Pathogen Databases (EuPathDB) (Aurrecoechea et al., 2010). Sequences for 3467 *B. canis* BcH-CHIPZ proteins were extracted from a Supplementary Excel spreadsheet created from the *B. canis* genome sequencing study (Eichenberger et al., 2017). Sequences for all 5460 *P. falciparum* (strain 3D7) and 8322 *T. gondii* (strain ME49) proteins were downloaded in a FASTA format from PlasmoDB (release 47) and ToxoDB (release 47), respectively, which are also database members of EuPathDB. Sequences from all five species were used in a FASTA format as primary input for each of the presented ML-SS methods.

### Rule-Based Method

SignalP 5.0 (Armenteros et al., 2019b) predicted the presence of SPs, TMHMM 2.0 (Krogh et al., 2001) predicted TMs, and PredGPI 1.0 (Pierleoni et al., 2008) predicted GPI-anchors. A protein is classified an exportome member if SignalP score ≥ 0.5, TMHMM predicted number of TMs = 0, and PredGPI predicted GPI FPrate (False Positive rate) ≥ 0.005; otherwise it is classified a non-exportome member. Note that GPI FPrate < 0.001 is highly probable, <0.005 is probable, <0.01 is weakly probable, and ≥0.01 is not GPI-anchored.

Two additional programs were used to compare the prediction outputs from SignalP and TMHMM: TargetP (Emanuelsson et al., 2007) (predicts SPs) and Phobius (Kall et al., 2004) (predicts SPs and TMs). A warning is given in the TMHMM 2.0 User’s guide that a predicted TM helix in the first 60 AAs of the N-terminal could be a SP. Note that 21 *B. bovis* proteins with a SignalP score ≥ 0.5 and TMHMM predicted number of TMs = 1 were classified an exportome, but only when the TM was predicted in the first 60 AAs. Phobius predictions supported that 17 of the 21 contained a SP but no TM.

### Training Input Sequences for Machine Learning

The 196 proteins representing the training positives (i.e., exportome members) were selected from the 3706 *B. bovis* T2Bo proteins following the rule-based bioinformatics approach. The 196 proteins representing the training negatives were randomly chosen using the Python random module (implements a Mersenne Twister (Matsumoto and Nishimura, 1998) as the core generator) from 3430 proteins predicted by the rule-based bioinformatics approach to be non-exportome members. The ML input training file for each method therefore consisted of 392 sequences representing the positives and negatives datasets. Supplementary Table 1 lists the rule-based selected proteins along with the SP, TMHMM, and GPI predicted characteristics on sheets ‘positives’ and ‘negatives’.

The program CD-HIT (cluster database at high identity with tolerance) (Li and Godzik, 2006) was used to determine whether any of the training and test sequences had 100% similarity (i.e., a check for redundant sequences). No identical sequences were detected, but four clusters of positive proteins had similarities >90% (see Supplementary Data 2). These proteins were assumed to be isoforms rather than the same proteins incorrectly assigned with unique IDs.

### Programs for Predicting Protein Secondary Structure Properties

Nine programs were selected that met the following requirements: standalone or at least had high-throughput processing capability, worked in a Linux environment, and generated an appropriate output from which SS properties could be extracted. The nine programs were Porter 5 (Torrisi et al., 2019), PSIPRED 4.0 (Jones, 1999; Buchan and Jones, 2019), NetsurfP 2.0 (Klausen et al., 2019), SSpro (Magnan and Baldi, 2014), SPOT-1D (Hanson et al., 2019), Spider3 (Heffernan et al., 2018), DeepCNF (Wang et al., 2016), MUFold (Fang et al., 2019, 2020), and Jpred 4 (Drozdetskiy et al., 2015). Predictors Jpred 4 and SPOT-1D have an 800 and MUFold a 700 AA length limit for input. Table 6 describes the algorithms used and the type of SS characteristics predicted by the nine programs. To enable high-throughput processing nine separate pipelines were created. Some predictors use different versions of the same Python modules and it was therefore not possible to create one generic pipeline suitable for all predictors. Furthermore, the outputs from these programs were different from each other and required the creation of nine Python scripts to extract, transform, and present the relevant SS in a consistent format, e.g., a series of H, E, or C for 3 class states and G, H, I, E, B, T, S, or C for 8 class states for each processed protein. The predictions varied considerably and therefore a consensus of the predicted classifications was created based on a majority rule approach applied at each amino acid. In the instance of a draw (e.g., due to a missing classification creating an equal number of classifications at a particular amino acid), predicted classifications were consecutively dropped from each predictor until a majority classification was achieved. The classifications from each predictor were dropped in the following order: DeepCNF, Spider3, Jpred 4, SSpro, NetsurfP, PSIPRED, SPOT-1D, and Porter 5. This order, from least to most accurate, was determined from evaluating the predictors (see Table 2).

**TABLE 6:** Publically available software for predicting protein secondary structure characteristics.

Program	Main algorithms	Evolutionary profile	Predicted SS characteristics
NetSurfP	Large Long Short-Term Memory (LSTM) network in a Bidirectional Recurrent Neural Network (BRNN) – trained on solved protein structures	PSSM created by PSIBLAST against UniRef90, and HMM profile created by HHblits given UniRef90	3 and 8 classes, ASA, CN, HSE, phi and psi
SPOT-1D	An ensemble of residual convolutional networks (ResNets) and Long-Short-Term Memory Cells in Bidirectional Recurrent Neural Networks (LSTM-BRNNs) with predicted contact maps input from SPOT-contact	PSSM created by PSIBLAST against UniRef90, and HMM profile created by HHblits given UniRef90	3 and 8 classes, ASA, CN, HSE, phi and psi
PSIPRED	Deep neural network architecture with two hidden layers, and with rectifier activations	PSSM created by PSIBLAST against UniRef90	3 classes
MUFOLD-SS and MUFOLDAngle	Variants of inception networks	PSSM created by PSIBLAST against UniRef90, and HMM profile created by HHblits given UniRef90	3 and 8 classes, phi and psi
Porter5	Ensembles of cascaded bidirectional recurrent neural networks and Convolutional Neural Networks (CNN)	PSSM created by PSIBLAST against UniRef90, and HMM profile created by HHblits given UniRef90	3 and 8 state
DeepCNF	Combines Conditional Neural Fields (CNF) and Deep Convolutional Neural Networks (DCNN)	PSSM created by PSIBLAST against UniRef90, and HMM profile created by HHblits given UniRef90	3 and 8 classes
SSPRO	An ensemble of 100 Bidirectional Recursive Neural Networks (BRNNs)	PSSM created by PSIBLAST against UniRef50	3 and 8 classes
SPIDER3 single	Long-Short-Term Memory Cells in Bidirectional Recurrent Neural Networks (LSTM-BRNNs)	Single-sequence-based prediction, i.e., no evolutionary profile used	3 and 8 classes, ASA, CN, HSE, phi and psi
Jpred4	Online tool using JNet algorithm	PSSM created by PSIBLAST against UniRef90	3 classes, ASA

*SS, protein secondary structure; 3 classes, 3 classes of secondary structure conformation; 8 classes, 8 classes of secondary structure conformation; phi, backbone torsion angle ϕ; psi, backbone torsion angle ψ; ASA, solvent-accessible surface area; CN, contact number; HSE, half-sphere exposure; PSSM, position specific substitution matrix; PSI-BLAST, Position-Specific Iterative Basic Local Alignment Search Tool; HMM, hidden Markov models; HHblits, a HMM-HMM-based iterative sequence search tool; UniRef50 and UniRef90, UniProt Reference Clusters from the UniProt Knowledgebase (UniProtKB) comprised of clustered sets of sequences with 50 or 90 sequence identity levels, respectively.*

### Machine Learning Algorithms

Six supervised ML algorithms were evaluated in this study for predicting exportome membership: adaptive boosting (AdaBoost), random forest (RF), *k*-nearest neighbour classifier, naive Bayes classifier, neural network, and support vector machines. AdaBoost and RF were the only two algorithms selected for final exportome predictions based on their superior 10-fold cross validation performances. AdaBoost (Freund and Schapire, 1997) and RF (Breiman, 2001) were implemented via the R functions *ada* (Friedman et al., 2000) and *randomForest*, respectively. Both R functions used at least two arguments: a data frame of numeric variables (i.e., a training dataset) and a numerical class vector, i.e., a vector representing the target label, which had two classes: 1 (positive) and 0 (negative). Each algorithm generates a probability that the binary classification is correct. Furthermore, both algorithms were used as an ensemble of classifiers, i.e., for each protein, classification probabilities from each algorithm in the ensemble were averaged to determine the final classification probability. Default ML parameters were used throughout except for the RF parameters ‘ntree’ and ‘mtry’ (changed to 300 and 3, respectively) and AdaBoost parameter ‘iter’ changed to 300.

### Creating Machine Learning Training Data With 3 and 8 Class State Predictions

**FIGURE 4:**
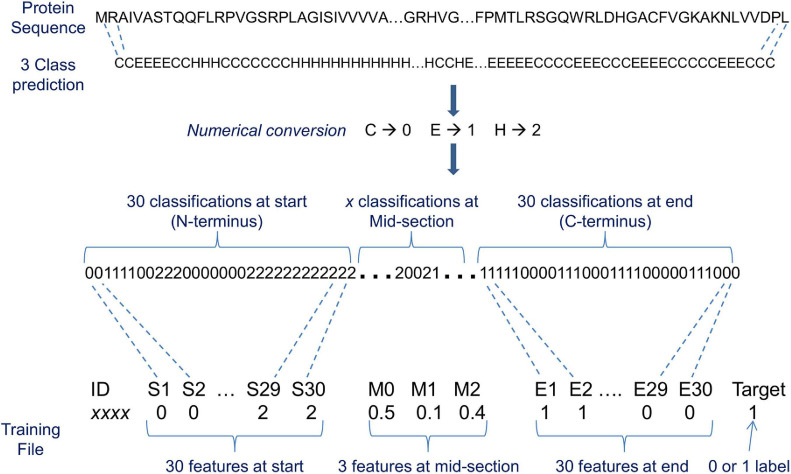
An illustration of a machine learning input representation for 3 classification state predictions of protein conformation. The 3 classes are helix, sheet, and coil designated H, E, and C, respectively. ‘*x*’ classifications at mid-section denotes that a varying number of classifications exist between a nominated start and end number of classifications (in this instance, 30) because the source proteins vary in length. S1, S2, etc. are the consecutively numbered feature names at the start, and E1, E2, etc. are the consecutively numbered feature names at the end. M0, M1, and M2 are the mid-section feature names that contain the total number of classifications for each structural class divided by the number of mid-section classifications (e.g., for 8 classification state predictions the mid-section feature names are M0, M1, M2, M3, M4, M5, M6, and M7). The target is what the machine learning (ML) algorithm attempts to predict, i.e., exportome (positive) or non-exportome (negative), and features are what the ML algorithm uses to help make the prediction. In this illustration, the total number of features used to represent varying length proteins is 63 per protein.

The training data protein sequences (196 negatives + 196 positives) were used as input to nine 3 class and seven 8 class predictors. Predicted classifications varied considerably between predictors as illustrated in Table 2. Therefore, a consensus of classifications was derived as previously described for each input protein (see Figure 2). The consensus classifications comprising letter characters were also converted to numerical values (C → 0, E → 1, H → 2 for 3 classes, and C → 0, S → 1, T → 2, B → 3, E → 4, I → 5, G → 6, H → 7 for 8 classes. Note that although each class was assigned a consistent value, the actual chosen value is arbitrary). Collectively, each consensus is of varying length due to a protein’s varying length. ML algorithms require a uniform set of features as input. The consensus classifications were therefore limited to various fixed length sections; such that the ML models were evaluated with different fixed length inputs. For instance, only a set number of classifications (features) from the consensus start and end, plus a set number of mid-section features were used as ML input (see Figure 4). Where the mid-section in this instance is either 3 or 8 features representing the total number of classifications for each particular structural class divided by the number of mid-section classifications, e.g., if 500 3 class classifications exist in a mid-section; whereby 250 are C, 50 are E, and 200 H; the three mid-section feature values are 0.5, 0.1, and 0.4.

Different variations of data input to the ML algorithms were evaluated using 10-fold cross validation. The variations evaluated include: start, middle, and end; start and middle; middle and end; start only; middle only; end only; start and remaining; start classes, middle classes, and end classes; start classes and middle classes; middle classes and end classes; start classes only; middle classes only; end classes only – where ‘start’ and ‘end’ is 30, 40, 50, 60, 75, or 100 classifications (equivalent to the number of AAs) measured from the N- or C-terminal, respectively; ‘middle’ is the remaining classifications between the two ends (the 3 or 8 classification structures are counted for the middle and divided by the number of mid-section classifications); ‘remaining’ is the remaining classifications after the start section and is calculated in the same way as ‘middle’, and ‘classes’ are 3 or 8 classification structure counts divided by the length of the relevant section. Normalisation or standardisation was also applied to all count features and the impact to the ML performances compared. The formulae used were Normalised *x* = (*x* – minimum *x*) / (maximum *x* – minimum *x*); and Standardised *x* = (*x* – μ) / σ. Where ‘*x*’ is the count value, and minimum and maximum relates to the minimum or maximum count value when considering the entire dataset (e.g., 3706 *B. bovis* proteins), and ‘μ’ is the sample mean and ‘σ’ is the standard deviation. As an additional test, predicted classifications were randomised and all the above variations evaluated, i.e., all input values were randomly shuffled using Python random module (with shuffle method). This test helps check whether the classification at a particular amino acid position makes a difference to the ML performance.

### Creating Machine Learning Training Data With Psi and Phi Angles

Predictors SPOT-1D, NetsurfP, and MUFold predict two sets of angles (psi and phi) between −180 and +180 for each amino acid as part of their output. The mean of the angles at each amino acid were determined. A challenge was how best to represent two sets of angles from proteins with varying length as a uniform set of values for ML input. The following input representations were evaluated with different fixed lengths (e.g., 40, 50, 60, and 75 AAs) from the N-terminal of the training sequences: (1) counting the number of AAs that fall into a particular angle range, e.g., a 30 step range – Range 1: ≤−150; Range 2: >−150 and ≤−120; Range 3: >−120 and ≤−90; Range 4: >−90 and ≤−60, etc. creating six ranges per angle type (psi or phi), and therefore 12 features in total; (2) counting the number of AAs that fall into a particular region on a ‘Psi vs. Phi’ angle plot (see Supplementary Figure 1). The plot is divided into four quadrants and then each quadrant is subdivided into regions. Each region represents a feature for ML input, whereby the feature value is the number of AAs within the region, e.g., if one squared region in the plot has a dimension of 30 – Region 1 in quadrant one is defined by Phi angles < 0 and ≤−30 *and* Psi angles > 0 and ≤30. There are 36 regions per quadrant and therefore 144 features in total for the entire plot; (3) using angles directly as the features, e.g., if the fixed length is 60 AAs then there are 60 features for phi and 60 for psi, and therefore 120 features in total; and (4) using angles directly as the features but combining the psi and phi angles as one feature by either multiplying or adding the two angles, e.g., if the fixed length is 60 AAs then there are 60 features for both phi and psi angles. Normalisation, standardisation, or no feature scaling was applied to all counts when evaluating representations. Note for representations #3 and #4, the assumption is that psi and phi angles recorded for amino acid #1 on a positive protein can be compared to the psi and phi angles recorded for amino acid #1 on a negative protein, and so on for each consecutive amino acid.

### Creating Machine Learning Training Data With Structural Properties ASA, CN, and HSE

Predictors SPOT-1D and Spider3 were used independently to predict for each amino acid in the input sequence, the structural properties of ASA, CN, and HSE for upper and down spheres. SPOT-1D and Spider3 save all three latter properties along with other data in one output file per protein with the extension ‘spot1d’ and ‘i1’, respectively. Each property (ASA, CN, HSE-upper, and HSE-down) were used in turn as features for ML input with different fixed lengths (e.g., 40, 50, 60, and 75 AAs) from the N-terminal of the training sequences.

### *Babesia bovis* Annotation Analysis

UniProtKB (Bateman et al., 2015) provides a heuristic measure of the annotation, although the curators claim they cannot define the ‘correct annotation’ for any given protein^1^. UniProtKB have assigned an annotation score from one to five to every protein, where five is considered the best-annotated entry (annotations with experimental evidence score higher than equivalent predicted/inferred annotations). With an understanding UniProtKB annotation scores are only a guideline of annotation quality, we checked scores for all *B. bovis* protein*s:* 89.1% scored 1, 10% scored 2, 0.87% scored 3, and 0.03% scored 4.

### Predicting the Presence of Transmembrane Domains Using Machine Learning and Secondary Structure Characteristics

We investigated the use of SS predictions, namely 3 and 8 classes, psi and phi angles, ASA, and HSE-upper to predict the presence of TM domains. The start and end of single or multiple TMs can occur anywhere within a protein sequence. For example, TMHMM predicts that 677 *B. bovis* T2Bo proteins contain at least one ranging to 22 TMs per protein starting anywhere from 2 AAs to 3405 AAs from the N-terminal. Furthermore, protein lengths of all 3706 *B. bovis* vary between 38 and 4820 AAs. These varying factors presented a challenge because ML requires a fixed set of values per protein for input. Supplementary Table 11 provides a breakdown of the number of *Babesia bovis* T2Bo proteins containing SPs and/or TM domains. For example, 575 of the 677 have no predicted SP. A ML training dataset comprising 539 positives and 539 negatives was created using predictions determined by TMHMM. Positives were proteins containing at least 1TM beyond 60 AAs from the N-terminal. The reasoning for ignoring the first 60 AAs was to prevent the impact of possible SPs. Negatives were proteins with no predicted TMs (see Supplementary Table 4).

Spider3 provided the secondary predictions. Different variations of fixed sets of values for ML input were evaluated using 10-fold cross validation. The variations evaluated included for each protein: counting the 3 or 8 classification structures; counting the number of AAs that fall into a particular psi or phi angle range; and counting the number of AAs that fall into a particular location on ‘Psi vs. Phi’ angle plot (see Supplementary Figure 1). All counts per protein were divided by the total number of characteristics counted (i.e., protein length – 60 AAs). Normalisation, standardisation, or no feature scaling was applied to all counts during evaluation.

### ML-SS Pipelines

The presented ML-SS methods have been implemented in five Linux pipelines. These pipelines consist of linked Python and Linux shell scripts, and R functions. The pipelines are designed to facilitate an automated, high-throughput computational approach to predict exportome membership probabilities. The five pipelines are named pipeline_ss (for 3 and 8 class predictions), pipeline_angles (for psi and phi angles predictions), pipeline_prop (for ASA and HSE-upper), pipeline_all (for 3 and 8 classes, psi and phi angles, ASA and HSE-upper) and pipeline_TM (for TM presence predictions). Four additional pipelines are also provided to conduct automated 10-fold cross validation of the ML-SS methods: pipeline_CV_ss, pipeline_CV_angles, pipeline_CV_prop, and pipeline_CV_TM. The pipelines were designed for a Linux operating system and have only been tested on Red Hat Enterprise Linux 7.7 but are expected to work on most Linux distributions. They are freely available at: https://github.com/goodswen/ML-SS_Methods. All pipelines are provided with a ReadMe file with instructions, and test and training data for *B. bovis* T2Bo. Note that the SS predictor programs are not packaged with the ML-SS pipelines and need to be downloaded and installed independently. The pipelines only require the raw output from the SS predictors.

## Data Availability Statement

All ML-SS methods presented in the article are freely available at: https://github.com/goodswen/ML-SS_Methods.

## Author Contributions

JE and SG contributed to the conception and methodology of the study. SG wrote the software, validated results, and performed the statistical analysis. JE and PK provided supervision. JE handled the project administration and funding acquisition. SG prepared the original draft. All authors contributed to manuscript revision, read, and approved the submitted version.

## Conflict of Interest

The authors declare that the research was conducted in the absence of any commercial or financial relationships that could be construed as a potential conflict of interest.

## Publisher’s Note

All claims expressed in this article are solely those of the authors and do not necessarily represent those of their affiliated organizations, or those of the publisher, the editors and the reviewers. Any product that may be evaluated in this article, or claim that may be made by its manufacturer, is not guaranteed or endorsed by the publisher.

## Footnotes

1 https://www.uniprot.org/help/annotation_score
