# Supplementary material for: Predicting Protein Therapeutic Candidates for Bovine Babesiosis Using Secondary Structure Properties and Machine Learning
Source: Front Genet. 2021 Jul 23;12:716132. doi: 10.3389/fgene.2021.716132 (PMC8343536; doi:10.3389/fgene.2021.716132)
Supplement: Supplementary file 1 [file Data_Sheet_1.PDF]

Supplementary Data S1

This document shows the machine learning performances of the exportome prediction methods (3 and 8 classes, psi and phi angles, ASA, and HSE-upper) when using various data input representations. The performances were evaluated using 10-fold cross validation on 196 positive and 196 negative proteins from *Babesia Bovis* T2Bo.

Description of performance measures:

TP = True Positive, **TN** = True Negative, **FP** = False Positive, **FN** = False Negative

Accuracy = (TP+TN)/(TP+FP+FN+TN) – how often the classifier is correct

Misclassification or Error Rate = (FP+FN)/(TP+FP+FN+TN) – how often the classifier is incorrect

True Positive Rate (TPR) or Sensitivity (SN) = TP/(TP + FN) – when the condition is positive, how often the classifier correctly predicts a positive condition

False Positive Rate (FPR) = FP/(FP + TN) – when the condition is negative, how often the classifier incorrectly predicts a positive condition

True Negative Rate (TNR) or Specificity (SP) = TN/(TN + FP) – when the condition is negative, how often the classifier correctly predicts a negative condition

Precision or Positive Predictive Value (PPV) = TP/(TP + FP) when the classifier predicts a positive condition, how often is the prediction correct

Negative Predictive Value (NPV) = TN/(TN + FN) – when the classifier predicts a negative condition, how often is the prediction correct.

Prevalence = how often the positive condition occurs in the sample

Description of machine learning algorithms:

ada = adaptive boosting (AdaBoost),

rf = random forest

Ensemble_score = for each protein, classification probabilities from ada and rf were averaged to determine the final classification probability.

8 class state predictions

First 25 AAs from N-Terminal + class count for remaining sequence (33 features)

Algorithm,Accuracy,Error Rate,Sensitivity,False Positive Rate,Specificity,Positive Predictive Value,Negative Predictive Value, Prevalence
ada,86.22,13.78,90.31,17.86,82.14,83.49,89.44,50.0
rf,86.48,13.52,91.84,18.88,81.12,82.95,90.86,50.0
average_ML_score,85.71,14.29,90.31,18.88,81.12,82.71,89.33,50.0

First 30 AAs from N-Terminal + class count for remaining sequence (38 features)

Algorithm,Accuracy,Error Rate,Sensitivity,False Positive Rate,Specificity,Positive Predictive Value,Negative Predictive Value, Prevalence
ada,85.89,14.11,88.12,16.34,83.66,84.36,87.56,50.0
rf,85.89,14.11,89.6,17.82,82.18,83.41,88.77,50.0
Ensemble_score,85.89,14.11,88.12,16.34,83.66,84.36,87.56,50.0

First 40 AAs from N-Terminal + class count for remaining sequence (48 features) (RF = 300 trees 3 splits)

Algorithm,Accuracy,Error Rate,Sensitivity,False Positive Rate,Specificity,Positive Predictive Value,Negative Predictive Value, Prevalence
ada,86.48,13.52,88.27,15.31,84.69,85.22,87.83,50.0
rf,85.71,14.29,87.24,15.82,84.18,84.65,86.84,50.0
Ensemble_score,86.73,13.27,87.76,14.29,85.71,86.0,87.5,50.0

First 40 AAs from N-Terminal + class count for remaining sequence (48 features) (Random shuffle)

Algorithm,Accuracy,Error Rate,Sensitivity,False Positive Rate,Specificity,Positive Predictive Value,Negative Predictive Value, Prevalence
ada,58.42,41.58,58.16,41.33,58.67,58.46,58.38,50.0
rf,59.95,40.05,60.71,40.82,59.18,59.8,60.1,50.0
Ensemble_score,57.91,42.09,57.65,41.84,58.16,57.95,57.87,50.0

First 40 AAs from N-Terminal + class count for remaining sequence (48 features) (RF = 300 trees 6 splits)

Algorithm,Accuracy,Error Rate,Sensitivity,False Positive Rate,Specificity,Positive Predictive Value,Negative Predictive Value, Prevalence
ada,86.14,13.86,87.13,14.85,85.15,85.44,86.87,50.0
rf,85.89,14.11,86.14,14.36,85.64,85.71,86.07,50.0
Ensemble_score,86.88,13.12,87.62,13.86,86.14,86.34,87.44,50.0

First 40 AAs from N-Terminal + class count for remaining sequence (48 features) (RF = 400 trees 3 splits)

Algorithm,Accuracy,Error Rate,Sensitivity,False Positive Rate,Specificity,Positive Predictive Value,Negative Predictive Value, Prevalence
ada,86.14,13.86,87.62,15.35,84.65,85.1,87.24,50.0

rf,85.15,14.85,86.63,16.34,83.66,84.13,86.22,50.0
Ensemble_score,86.63,13.37,87.62,14.36,85.64,85.92,87.37,50.0

First 40 AAs from N-Terminal + class count for remaining sequence (48 features) (RF = 500 trees 3 splits)

Algorithm,Accuracy,Error Rate,Sensitivity,False Positive Rate,Specificity,Positive Predictive Value,Negative Predictive Value, Prevalence
ada,86.63,13.37,88.12,14.85,85.15,85.58,87.76,50.0
rf,85.89,14.11,87.62,15.84,84.16,84.69,87.18,50.0
Ensemble_score,86.63,13.37,88.12,14.85,85.15,85.58,87.76,50.0

First 50 AAs from N-Terminal + class count for remaining sequence (58 features)

Algorithm,Accuracy,Error Rate,Sensitivity,False Positive Rate,Specificity,Positive Predictive Value,Negative Predictive Value, Prevalence
ada,84.9,15.1,87.13,17.33,82.67,83.41,86.53,50.0
rf,85.64,14.36,87.13,15.84,84.16,84.62,86.73,50.0
Ensemble_score,85.64,14.36,88.12,16.83,83.17,83.96,87.5,50.0

First 60 AAs from N-Terminal + class count for remaining sequence (68 features)

Algorithm,Accuracy,Error Rate,Sensitivity,False Positive Rate,Specificity,Positive Predictive Value,Negative Predictive Value, Prevalence
ada,86.32,13.68,88.56,15.92,84.08,84.76,88.02,50.0
rf,84.58,15.42,87.06,17.91,82.09,82.94,86.39,50.0
Ensemble_score,85.57,14.43,87.06,15.92,84.08,84.54,86.67,50.0

First 100 AAs from N-Terminal + class count for remaining sequence (108 features)

Algorithm,Accuracy,Error Rate,Sensitivity,False Positive Rate,Specificity,Positive Predictive Value,Negative Predictive Value, Prevalence
ada,84.2,15.8,85.49,17.1,82.9,83.33,85.11,50.0
rf,83.42,16.58,84.46,17.62,82.38,82.74,84.13,50.0
Ensemble_score,84.72,15.28,86.01,16.58,83.42,83.84,85.64,50.0

First 130 AAs from N-Terminal + class count for remaining sequence (138 features)

Algorithm,Accuracy,Error Rate,Sensitivity,False Positive Rate,Specificity,Positive Predictive Value,Negative Predictive Value, Prevalence
ada,84.64,15.36,85.47,16.2,83.8,84.07,85.23,50.0
rf,82.4,17.6,85.47,20.67,79.33,80.53,84.52,50.0
Ensemble_score,84.92,15.08,86.03,16.2,83.8,84.15,85.71,50.0

First 40 AAs from N-Terminal + class count for remaining sequence (48 features) (excludes DeepCNF and Spider3)

Algorithm,Accuracy,Error Rate,Sensitivity,False Positive Rate,Specificity,Positive Predictive Value,Negative Predictive Value, Prevalence

ada,85.15,14.85,85.64,15.35,84.65,84.8,85.5,50.0
rf,85.89,14.11,86.14,14.36,85.64,85.71,86.07,50.0
Ensemble_score,84.9,15.1,86.14,16.34,83.66,84.06,85.79,50.0

First 40 AAs from N-Terminal + class count for remaining sequence (48 features) (SPOT-1D only)

Algorithm,Accuracy,Error Rate,Sensitivity,False Positive Rate,Specificity,Positive Predictive Value,Negative Predictive Value, Prevalence
ada,82.92,17.08,82.67,16.83,83.17,83.08,82.76,50.0
rf,83.91,16.09,86.63,18.81,81.19,82.16,85.86,50.0
Ensemble_score,82.43,17.57,82.67,17.82,82.18,82.27,82.59,50.0

First 40 AAs from N-Terminal + class count for mid-section + end 40 AAs (88 features)

Algorithm,Accuracy,Error Rate,Sensitivity,False Positive Rate,Specificity,Positive Predictive Value,Negative Predictive Value, Prevalence
ada,84.1,15.9,85.64,17.44,82.56,83.08,85.19,50.0
rf,83.85,16.15,85.13,17.44,82.56,83.0,84.74,50.0
Ensemble_score,84.36,15.64,85.64,16.92,83.08,83.5,85.26,50.0

3 class state predictions

First 25 AAs from N-Terminal + class count for remaining sequence (28 features)

Algorithm,Accuracy,Error Rate,Sensitivity,False Positive Rate,Specificity,Positive Predictive Value,Negative Predictive Value, Prevalence
ada,83.67,16.33,85.71,18.37,81.63,82.35,85.11,50.0
rf,85.46,14.54,89.29,18.37,81.63,82.94,88.4,50.0
average_ML_score,84.69,15.31,87.24,17.86,82.14,83.01,86.56,50.0

First 30 AAs from N-Terminal + class count for remaining sequence (33 features)

Algorithm,Accuracy,Error Rate,Sensitivity,False Positive Rate,Specificity,Positive Predictive Value,Negative Predictive Value, Prevalence
ada,87.13,12.87,90.59,16.34,83.66,84.72,89.89,50.0
rf,84.9,15.1,86.63,16.83,83.17,83.73,86.15,50.0
Ensemble_score,87.13,12.87,90.59,16.34,83.66,84.72,89.89,50.0

First 40 AAs from N-Terminal + class count for remaining sequence (43 features)

Algorithm,Accuracy,Error Rate,Sensitivity,False Positive Rate,Specificity,Positive Predictive Value,Negative Predictive Value, Prevalence
ada,86.73,13.27,88.27,14.8,85.2,85.64,87.89,50.0
rf,87.24,12.76,89.29,14.8,85.2,85.78,88.83,50.0
Ensemble_score,86.99,13.01,87.76,13.78,86.22,86.43,87.56,50.0

First 40 AAs from N-Terminal + class count for remaining sequence (43 features) (Random shuffle)

Algorithm,Accuracy,Error Rate,Sensitivity,False Positive Rate,Specificity,Positive Predictive Value,Negative Predictive Value, Prevalence
ada,56.89,43.11,55.61,41.84,58.16,57.07,56.72,50.0
rf,54.34,45.66,54.59,45.92,54.08,54.31,54.36,50.0

Ensemble_score,56.12,43.88,55.1,42.86,57.14,56.25,56.0,50.0

First 50 AAs from N-Terminal + class count for remaining sequence (53 features)

Algorithm,Accuracy,Error Rate,Sensitivity,False Positive Rate,Specificity,Positive Predictive Value,Negative Predictive Value, Prevalence
ada,87.13,12.87,90.59,16.34,83.66,84.72,89.89,50.0
rf,84.9,15.1,86.63,16.83,83.17,83.73,86.15,50.0
Ensemble_score,87.13,12.87,90.59,16.34,83.66,84.72,89.89,50.0

First 70 AAs from N-Terminal + class count for remaining sequence (73 features)

Algorithm,Accuracy,Error Rate,Sensitivity,False Positive Rate,Specificity,Positive Predictive Value,Negative Predictive Value, Prevalence
ada,86.25,13.75,89.0,16.5,83.5,84.36,88.36,50.0
rf,85.75,14.25,88.0,16.5,83.5,84.21,87.43,50.0
Ensemble_score,86.5,13.5,89.0,16.0,84.0,84.76,88.42,50.0

class counts (xnorm) (3 features)

Algorithm,Accuracy,Error Rate,Sensitivity,False Positive Rate,Specificity,Positive Predictive Value,Negative Predictive Value, Prevalence
ada,76.24,23.76,80.2,27.72,72.28,74.31,78.49,50.0
rf,74.75,25.25,82.18,32.67,67.33,71.55,79.07,50.0
Ensemble_score,75.74,24.26,80.69,29.21,70.79,73.42,78.57,50.0

Phi and PSI Angles

using Angles directly from first 40 AAs from N-Terminal (e.g., 40 Psi + 40 Phi features = 80 features) (no feature scaling)

Algorithm,Accuracy,Error Rate,Sensitivity,False Positive Rate,Specificity,Positive Predictive Value,Negative Predictive Value, Prevalence
ada,83.42,16.58,84.65,17.82,82.18,82.61,84.26,50.0
rf,84.41,15.59,79.7,10.89,89.11,87.98,81.45,50.0
Ensemble_score,83.91,16.09,83.66,15.84,84.16,84.08,83.74,50.0

using Angles directly from first 40 AAs from N-Terminal (e.g., 40 Psi + 40 Phi features = 80 features) (normalisation)

Algorithm,Accuracy,Error Rate,Sensitivity,False Positive Rate,Specificity,Positive Predictive Value,Negative Predictive Value, Prevalence
ada,86.73,13.27,86.73,13.27,86.73,86.73,86.73,50.0
rf,84.95,15.05,80.1,10.2,89.8,88.7,81.86,50.0
Ensemble_score,86.73,13.27,86.22,12.76,87.24,87.11,86.36,50.0

using Angles directly from first 40 AAs from N-Terminal (e.g., 40 Psi + 40 Phi features = 80 features) (normalisation) (random shuffle)

Algorithm,Accuracy,Error Rate,Sensitivity,False Positive Rate,Specificity,Positive Predictive Value,Negative Predictive Value, Prevalence

ada,55.1,44.9,59.69,49.49,50.51,54.67,55.62,50.0
rf,56.63,43.37,61.22,47.96,52.04,56.07,57.3,50.0
Ensemble_score,54.85,45.15,59.18,49.49,50.51,54.46,55.31,50.0

using Angles directly from first 30 AAs from N-Terminal (e.g., 30 Psi + 30 Phi features = 60 features) (standardisation)

Algorithm,Accuracy,Error Rate,Sensitivity,False Positive Rate,Specificity,Positive Predictive Value,Negative Predictive Value, Prevalence
ada,84.65,15.35,84.65,15.35,84.65,84.65,84.65,50.0
rf,84.65,15.35,79.21,9.9,90.1,88.89,81.25,50.0
Ensemble_score,84.9,15.1,84.65,14.85,85.15,85.07,84.73,50.0

using Angles directly from first 40 AAs from N-Terminal (e.g., 40 Psi + 40 Phi features = 80 features) (standardisation)

Algorithm,Accuracy,Error Rate,Sensitivity,False Positive Rate,Specificity,Positive Predictive Value,Negative Predictive Value, Prevalence
ada,86.73,13.27,86.22,12.76,87.24,87.11,86.36,50.0
rf,84.95,15.05,80.1,10.2,89.8,88.7,81.86,50.0
Ensemble_score,86.73,13.27,85.2,11.73,88.27,87.89,85.64,50.0

using Angles directly from first 50 AAs from N-Terminal (e.g., 50 Psi + 50 Phi features = 100 features) (standardisation)

Algorithm,Accuracy,Error Rate,Sensitivity,False Positive Rate,Specificity,Positive Predictive Value,Negative Predictive Value, Prevalence
ada,83.91,16.09,83.17,15.35,84.65,84.42,83.41,50.0
rf,83.91,16.09,78.71,10.89,89.11,87.85,80.72,50.0
Ensemble_score,84.41,15.59,83.17,14.36,85.64,85.28,83.57,50.0

using Angles directly from first 60 AAs from N-Terminal (e.g., 60 Psi + 60 Phi features = 120 features) (no feature scaling)

Algorithm,Accuracy,Error Rate,Sensitivity,False Positive Rate,Specificity,Positive Predictive Value,Negative Predictive Value, Prevalence
ada,84.83,15.17,83.08,13.43,86.57,86.08,83.65,50.0
rf,84.08,15.92,79.1,10.95,89.05,87.85,81.0,50.0
Ensemble_score,84.83,15.17,82.59,12.94,87.06,86.46,83.33,50.0

using Angles directly from first 60 AAs from N-Terminal (e.g., 60 Psi + 60 Phi features = 120 features) (normalisation)

Algorithm,Accuracy,Error Rate,Sensitivity,False Positive Rate,Specificity,Positive Predictive Value,Negative Predictive Value, Prevalence
ada,85.07,14.93,84.08,13.93,86.07,85.79,84.39,50.0
rf,83.83,16.17,79.1,11.44,88.56,87.36,80.91,50.0
Ensemble_score,84.83,15.17,84.08,14.43,85.57,85.35,84.31,50.0

using Angles directly from first 60 AAs from N-Terminal (e.g., 60 Psi + 60 Phi features = 120 features) (standardisation)

Algorithm,Accuracy,Error Rate,Sensitivity,False Positive Rate,Specificity,Positive Predictive Value,Negative Predictive Value, Prevalence
ada,84.33,15.67,83.08,14.43,85.57,85.2,83.5,50.0
rf,83.58,16.42,78.61,11.44,88.56,87.29,80.54,50.0
Ensemble_score,84.33,15.67,82.09,13.43,86.57,85.94,82.86,50.0

using Angles directly from first 40 AAs from N-Terminal (e.g., 40 Psi + 40 Phi features = 80 features)

Algorithm,Accuracy,Error Rate,Sensitivity,False Positive Rate,Specificity,Positive Predictive Value,Negative Predictive Value, Prevalence
ada,84.22,15.78,82.89,14.44,85.56,85.16,83.33,50.0
rf,83.96,16.04,78.61,10.7,89.3,88.02,80.68,50.0
Ensemble_score,85.29,14.71,83.42,12.83,87.17,86.67,84.02,50.0

using Angles directly from first 50 AAs from N-Terminal (e.g., 50 Psi + 50 Phi features = 100 features)

Algorithm,Accuracy,Error Rate,Sensitivity,False Positive Rate,Specificity,Positive Predictive Value,Negative Predictive Value, Prevalence
ada,84.49,15.51,82.89,13.9,86.1,85.64,83.42,50.0
rf,83.69,16.31,78.07,10.7,89.3,87.95,80.29,50.0
Ensemble_score,84.76,15.24,81.82,12.3,87.7,86.93,82.83,50.0

counting the number of AAs that fall into a particular region on a 'Psi vs Phi' angle plot (Step 30 = 144 features)

Algorithm,Accuracy,Error Rate,Sensitivity,False Positive Rate,Specificity,Positive Predictive Value,Negative Predictive Value, Prevalence
ada,80.75,19.25,82.89,21.39,78.61,79.49,82.12,50.0
rf,69.52,30.48,96.26,57.22,42.78,62.72,91.95,50.0
Ensemble_score,80.75,19.25,83.96,22.46,77.54,78.89,82.86,50.0

counting the number of AAs that fall into a particular region on a 'Psi vs Phi' angle plot (Step 60 – 36 features)

Algorithm,Accuracy,Error Rate,Sensitivity,False Positive Rate,Specificity,Positive Predictive Value,Negative Predictive Value, Prevalence
ada,67.08,32.92,66.83,32.67,67.33,67.16,67.0,50.0
rf,67.82,32.18,67.82,32.18,67.82,67.82,67.82,50.0
Ensemble_score,68.56,31.44,67.82,30.69,69.31,68.84,68.29,50.0

counting the number of AAs that fall into a particular angle range (Step 30 – 12 features) (no feature scaling)

Algorithm,Accuracy,Error Rate,Sensitivity,False Positive Rate,Specificity,Positive Predictive Value,Negative Predictive Value, Prevalence
ada,78.07,21.93,77.54,21.39,78.61,78.38,77.78,50.0
rf,78.61,21.39,78.61,21.39,78.61,78.61,78.61,50.0
Ensemble_score,79.41,20.59,79.14,20.32,79.68,79.57,79.26,50.0

counting the number of AAs that fall into a particular angle range (Step 60 – 6 features) (normalisation)

Algorithm,Accuracy,Error Rate,Sensitivity,False Positive Rate,Specificity,Positive Predictive Value,Negative Predictive Value, Prevalence
ada,68.56,31.44,68.81,31.68,68.32,68.47,68.66,50.0
rf,67.57,32.43,67.82,32.67,67.33,67.49,67.66,50.0
Ensemble_score,68.07,31.93,67.33,31.19,68.81,68.34,67.8,50.0

using angles directly as the features but combining the psi and phi angles as one feature by **adding** the two angles. First 40 AAs from N-Terminal (e.g., 40 features)

Algorithm,Accuracy,Error Rate,Sensitivity,False Positive Rate,Specificity,Positive Predictive Value,Negative Predictive Value, Prevalence
ada,82.35,17.65,82.35,17.65,82.35,82.35,82.35,50.0
rf,83.96,16.04,79.14,11.23,88.77,87.57,80.98,50.0
Ensemble_score,83.69,16.31,82.89,15.51,84.49,84.24,83.16,50.0

using angles directly as the features but combining the psi and phi angles as one feature by multiplying the two angles. First 40 AAs from N-Terminal (e.g., 40 features)

Algorithm,Accuracy,Error Rate,Sensitivity,False Positive Rate,Specificity,Positive Predictive Value,Negative Predictive Value, Prevalence
ada,81.55,18.45,80.21,17.11,82.89,82.42,80.73,50.0
rf,81.82,18.18,78.61,14.97,85.03,84.0,79.9,50.0
Ensemble_score,81.28,18.72,79.68,17.11,82.89,82.32,80.31,50.0

using angles directly as the features but combining the psi and phi angles as one feature by multiplying the two angles. First 50 AAs from N-Terminal (e.g., 50 features)

Algorithm,Accuracy,Error Rate,Sensitivity,False Positive Rate,Specificity,Positive Predictive Value,Negative Predictive Value, Prevalence
ada,79.95,20.05,78.61,18.72,81.28,80.77,79.17,50.0
rf,81.82,18.18,78.61,14.97,85.03,84.0,79.9,50.0
Ensemble_score,80.75,19.25,79.68,18.18,81.82,81.42,80.1,50.0

<u>Exposure structural properties </u>(using Spider3 input only)

using **ASA** values from first 40 AAs from N-Terminal (40 features) (standardisation)
Algorithm,Accuracy,Error Rate,Sensitivity,False Positive Rate,Specificity,Positive Predictive Value,Negative Predictive Value, Prevalence
ada,90.31,9.69,93.37,12.76,87.24,87.98,92.93,50.0
rf,88.78,11.22,90.82,13.27,86.73,87.25,90.43,50.0
Ensemble_score,90.31,9.69,93.37,12.76,87.24,87.98,92.93,50.0

using **ASA** values from first 40 AAs from N-Terminal (40 features) (xnorm)
Algorithm,Accuracy,Error Rate,Sensitivity,False Positive Rate,Specificity,Positive Predictive Value,Negative Predictive Value, Prevalence
ada,90.05,9.95,92.86,12.76,87.24,87.92,92.43,50.0
rf,90.05,9.95,92.86,12.76,87.24,87.92,92.43,50.0
Ensemble_score,90.56,9.44,93.88,12.76,87.24,88.04,93.44,50.0

using **ASA** values from first 40 AAs from N-Terminal (40 features) (No feature scaling)

Algorithm,Accuracy,Error Rate,Sensitivity,False Positive Rate,Specificity,Positive Predictive Value,Negative Predictive Value, Prevalence
ada,90.05,9.95,92.86,12.76,87.24,87.92,92.43,50.0
rf,89.54,10.46,91.33,12.24,87.76,88.18,91.01,50.0
Ensemble_score,90.31,9.69,93.37,12.76,87.24,87.98,92.93,50.0

using **ASA** values from first 25 AAs from N-Terminal (25 features) (No feature scaling)

Algorithm,Accuracy,Error Rate,Sensitivity,False Positive Rate,Specificity,Positive Predictive Value,Negative Predictive Value, Prevalence
ada,89.03,10.97,91.84,13.78,86.22,86.96,91.35,50.0
rf,87.24,12.76,88.78,14.29,85.71,86.14,88.42,50.0
average_ML_score,89.03,10.97,91.33,13.27,86.73,87.32,90.91,50.0

using **ASA** values from first 40 AAs from N-Terminal (40 features) (No feature scaling) (random shuffle)

Algorithm,Accuracy,Error Rate,Sensitivity,False Positive Rate,Specificity,Positive Predictive Value,Negative Predictive Value, Prevalence
ada,75.0,25.0,79.08,29.08,70.92,73.11,77.22,50.0
rf,75.77,24.23,81.12,29.59,70.41,73.27,78.86,50.0
average_ML_score,75.0,25.0,79.08,29.08,70.92,73.11,77.22,50.0

using **ASA** values from first 40 AAs from N-Terminal (40 features) (No feature scaling) (random sample)

Algorithm,Accuracy,Error Rate,Sensitivity,False Positive Rate,Specificity,Positive Predictive Value,Negative Predictive Value, Prevalence
ada,73.47,26.53,76.53,29.59,70.41,72.12,75.0,50.0
rf,75.26,24.74,81.12,30.61,69.39,72.6,78.61,50.0
average_ML_score,73.21,26.79,76.02,29.59,70.41,71.98,74.59,50.0

using **HSE_upper** values from first 40 AAs from N-Terminal (40 features) (no feature scaling)

Algorithm,Accuracy,Error Rate,Sensitivity,False Positive Rate,Specificity,Positive Predictive Value,Negative Predictive Value, Prevalence
ada,89.54,10.46,92.86,13.78,86.22,87.08,92.35,50.0
rf,91.84,8.16,93.37,9.69,90.31,90.59,93.16,50.0
Ensemble_score,90.31,9.69,93.37,12.76,87.24,87.98,92.93,50.0

using **HSE_upper** values from first 40 AAs from N-Terminal (40 features) (no feature scaling) (random shuffle)

Algorithm,Accuracy,Error Rate,Sensitivity,False Positive Rate,Specificity,Positive Predictive Value,Negative Predictive Value, Prevalence
ada,73.72,26.28,77.55,30.1,69.9,72.04,75.69,50.0
rf,74.49,25.51,79.08,30.1,69.9,72.43,76.97,50.0
Ensemble_score,73.47,26.53,77.04,30.1,69.9,71.9,75.27,50.0

using **HSE_upper** values from first 40 AAs from N-Terminal (40 features) (no feature scaling) (random sample)

Algorithm,Accuracy,Error Rate,Sensitivity,False Positive Rate,Specificity,Positive Predictive Value,Negative Predictive Value, Prevalence
ada,72.7,27.3,74.49,29.08,70.92,71.92,73.54,50.0
rf,75.51,24.49,78.57,27.55,72.45,74.04,77.17,50.0
average_ML_score,71.68,28.32,71.94,28.57,71.43,71.57,71.79,50.0

using HSE_upper values from first 40 AAs from N-Terminal (40 features) (normalisation)

Algorithm,Accuracy,Error Rate,Sensitivity,False Positive Rate,Specificity,Positive Predictive Value,Negative Predictive Value, Prevalence
ada,90.31,9.69,93.37,12.76,87.24,87.98,92.93,50.0
rf,91.58,8.42,92.86,9.69,90.31,90.55,92.67,50.0
Ensemble_score,90.82,9.18,93.37,11.73,88.27,88.83,93.01,50.0

using HSE_upper values from first 40 AAs from N-Terminal (40 features) (standardisation)

Algorithm,Accuracy,Error Rate,Sensitivity,False Positive Rate,Specificity,Positive Predictive Value,Negative Predictive Value, Prevalence
ada,90.31,9.69,93.37,12.76,87.24,87.98,92.93,50.0
rf,91.58,8.42,93.37,10.2,89.8,90.15,93.12,50.0
Ensemble_score,90.56,9.44,93.88,12.76,87.24,88.04,93.44,50.0

using HSE_down values from first 40 AAs from N-Terminal (40 features) (none)

Algorithm,Accuracy,Error Rate,Sensitivity,False Positive Rate,Specificity,Positive Predictive Value,Negative Predictive Value, Prevalence
ada,92.09,7.91,94.9,10.71,89.29,89.86,94.59,50.0
rf,91.33,8.67,92.86,10.2,89.8,90.1,92.63,50.0
Ensemble_score,92.09,7.91,94.9,10.71,89.29,89.86,94.59,50.0

using CN values from first 40 AAs from N-Terminal (40 features) (no feature scaling)

Algorithm,Accuracy,Error Rate,Sensitivity,False Positive Rate,Specificity,Positive Predictive Value,Negative Predictive Value, Prevalence
ada,91.33,8.67,94.39,11.73,88.27,88.94,94.02,50.0
rf,90.56,9.44,92.35,11.22,88.78,89.16,92.06,50.0
Ensemble_score,91.33,8.67,93.88,11.22,88.78,89.32,93.55,50.0

<u>Exposure structural properties</u> (using SPOT-1D input only)

using HSE_upper values from first 40 AAs from N-Terminal (40 features) (no feature scaling)

Algorithm,Accuracy,Error Rate,Sensitivity,False Positive Rate,Specificity,Positive Predictive Value,Negative Predictive Value, Prevalence
ada,86.22,13.78,84.69,12.24,87.76,87.37,85.15,50.0
rf,86.99,13.01,82.14,8.16,91.84,90.96,83.72,50.0
Ensemble_score,86.48,13.52,84.69,11.73,88.27,87.83,85.22,50.0

using HSE_upper values from first 40 AAs from N-Terminal (40 features) (xnorm)

Algorithm,Accuracy,Error Rate,Sensitivity,False Positive Rate,Specificity,Positive Predictive Value,Negative Predictive Value, Prevalence

ada,86.39,13.61,84.65,11.88,88.12,87.69,85.17,50.0
rf,86.63,13.37,82.67,9.41,90.59,89.78,83.94,50.0
Ensemble_score,86.39,13.61,84.65,11.88,88.12,87.69,85.17,50.0

using HSE_upper values from first 40 AAs from N-Terminal (40 features) (standard)

Algorithm,Accuracy,Error Rate,Sensitivity,False Positive Rate,Specificity,Positive Predictive Value,Negative Predictive Value, Prevalence
ada,86.14,13.86,85.15,12.87,87.13,86.87,85.44,50.0
rf,86.63,13.37,82.67,9.41,90.59,89.78,83.94,50.0
Ensemble_score,85.89,14.11,83.66,11.88,88.12,87.56,84.36,50.0

using asa values from first 40 AAs from N-Terminal (40 features) (no feature scaling)

Algorithm,Accuracy,Error Rate,Sensitivity,False Positive Rate,Specificity,Positive Predictive Value,Negative Predictive Value, Prevalence
ada,87.5,12.5,86.22,11.22,88.78,88.48,86.57,50.0
rf,86.22,13.78,81.63,9.18,90.82,89.89,83.18,50.0
Ensemble_score,87.76,12.24,86.22,10.71,89.29,88.95,86.63,50.0

using asa values from first 40 AAs from N-Terminal (40 features) (xnorm)

Algorithm,Accuracy,Error Rate,Sensitivity,False Positive Rate,Specificity,Positive Predictive Value,Negative Predictive Value, Prevalence
ada,87.13,12.87,88.61,14.36,85.64,86.06,88.27,50.0
rf,86.39,13.61,81.68,8.91,91.09,90.16,83.26,50.0
Ensemble_score,86.14,13.86,86.63,14.36,85.64,85.78,86.5,50.0

using asa values from first 40 AAs from N-Terminal (40 features) (standard)

Algorithm,Accuracy,Error Rate,Sensitivity,False Positive Rate,Specificity,Positive Predictive Value,Negative Predictive Value, Prevalence
ada,87.38,12.62,87.62,12.87,87.13,87.19,87.56,50.0
rf,86.39,13.61,81.68,8.91,91.09,90.16,83.26,50.0
Ensemble_score,86.88,13.12,86.14,12.38,87.62,87.44,86.34,50.0

using asa values from first 50 AAs from N-Terminal (50 features) (none)

Algorithm,Accuracy,Error Rate,Sensitivity,False Positive Rate,Specificity,Positive Predictive Value,Negative Predictive Value, Prevalence
ada,87.13,12.87,87.13,12.87,87.13,87.13,87.13,50.0
rf,85.89,14.11,81.19,9.41,90.59,89.62,82.81,50.0
Ensemble_score,86.14,13.86,85.15,12.87,87.13,86.87,85.44,50.0

using CN values from first 40 AAs from N-Terminal (40 features) (no feature scaling)

Algorithm,Accuracy,Error Rate,Sensitivity,False Positive Rate,Specificity,Positive Predictive Value,Negative Predictive Value, Prevalence
ada,83.17,16.83,84.65,18.32,81.68,82.21,84.18,50.0
rf,84.65,15.35,81.68,12.38,87.62,86.84,82.71,50.0
Ensemble_score,83.66,16.34,83.66,16.34,83.66,83.66,83.66,50.0

using HSE_down values from first 40 AAs from N-Terminal (40 features) (no feature scaling)
Algorithm,Accuracy,Error Rate,Sensitivity,False Positive Rate,Specificity,Positive Predictive Value,Negative Predictive Value, Prevalence

ada,83.17,16.83,85.64,19.31,80.69,81.6,84.9,50.0
rf,84.16,15.84,82.67,14.36,85.64,85.2,83.17,50.0
Ensemble_score,83.91,16.09,86.14,18.32,81.68,82.46,85.49,50.0
