# Supplementary material for: Predicting Protein Therapeutic Candidates for Bovine Babesiosis Using Secondary Structure Properties and Machine Learning
Source: Front Genet. 2021 Jul 23;12:716132. doi: 10.3389/fgene.2021.716132 (PMC8343536; doi:10.3389/fgene.2021.716132)
Supplement: Supplementary file 2 [file Data_Sheet_2.PDF]

Supplementary Data S2: CD-HIT results

### Positive (exportome) proteins

Program: CD-HIT, V4.8.1 (+OpenMP)

Command: ./cd-hit -i positives_196.fa -o cdhit_positives_196

==

Output

----

total seq: 196

longest and shortest : 1708 and 65

Total letters: 74541

Sequences have been sorted

Approximated minimal memory consumption:

Sequence : 0M

Buffer : 1 X 10M = 10M

Table : 1 X 65M = 65M

Miscellaneous : 0M

Total : 76M

Table limit with the given memory limit:

Max number of representatives: 1343387

Max number of word counting entries: 90461699

comparing sequences from 0 to 196

196 finished 191 clusters

Approximated maximum memory consumption: 76M

writing new database

writing clustering information

program completed !

Total CPU time 0.06

##

sequence identity threshold, default 0.9

>Cluster 0

0 1708aa, >BBOV_IV005270... *

>Cluster 1

0 1536aa, >BBOV_IV011430... *

>Cluster 2

0 1089aa, >BBOV_I004210... *

>Cluster 3

0 1069aa, >BBOV_III009720... *

>Cluster 4

0 1052aa, >BBOV_III007200... *

>Cluster 5

0 1014aa, >BBOV_III001220... *

>Cluster 6

0 969aa, >BBOV_IV007480... *

>Cluster 7

0 865aa, >BBOV_III003260... *

>Cluster 8

0 817aa, >BBOV_IV004710... *

>Cluster 9

0 814aa, >BBOV_III004200... *

>Cluster 10

0 810aa, >BBOV_IV004200... *

>Cluster 11

0 800aa, >BBOV_II006200... *

>Cluster 12

0 795aa, >BBOV_IV008400... *

>Cluster 13

0 795aa, >BBOV_III007380... *

>Cluster 14

0 790aa, >BBOV_I001080... *

>Cluster 15

0 788aa, >BBOV_IV002260... *

>Cluster 16

0 784aa, >BBOV_IV002270... *

>Cluster 17

0 781aa, >BBOV_IV007620... *

>Cluster 18

0 778aa, >BBOV_IV009240... *

>Cluster 19

0 772aa, >BBOV_II006930... *

>Cluster 20

0 711aa, >BBOV_III003040... *

>Cluster 21

0 665aa, >BBOV_III005960... *

>Cluster 22

0 652aa, >BBOV_III007800... *

>Cluster 23

0 651aa, >BBOV_IV007010... *

>Cluster 24

0 629aa, >BBOV_II004000... *

>Cluster 25

0 627aa, >BBOV_III001600... *

>Cluster 26

0 622aa, >BBOV_IV004810... *

>Cluster 27

0 616aa, >BBOV_III007900... *

>Cluster 28

0 613aa, >BBOV_II004280... *

>Cluster 29

0 613aa, >BBOV_III004080... *

>Cluster 30

0 611aa, >BBOV_IV001950... *

>Cluster 31

0 601aa, >BBOV_IV010640... *

>Cluster 32

0 598aa, >BBOV_II006600... *

>Cluster 33

0 593aa, >BBOV_II002190... *

>Cluster 34

0 575aa, >BBOV_IV004070... *

>Cluster 35

0 565aa, >BBOV_IV009870... *

>Cluster 36

0 559aa, >BBOV_II001970... *

>Cluster 37

0 548aa, >BBOV_III005180... *

>Cluster 38

0 534aa, >BBOV_I000990... *

>Cluster 39

0 531aa, >BBOV_IV001070... *

>Cluster 40

0 529aa, >BBOV_I001010... *

>Cluster 41

0 517aa, >BBOV_IV007750... *

>Cluster 42

0 509aa, >BBOV_III009440... *

>Cluster 43

0 501aa, >BBOV_I000160... *

>Cluster 44

0 480aa, >BBOV_II000970... *

>Cluster 45

0 463aa, >BBOV_IV002330... *

>Cluster 46

0 459aa, >BBOV_IV006390... *

>Cluster 47

0 449aa, >BBOV_IV011940... *

>Cluster 48

0 438aa, >BBOV_IV003980... *

>Cluster 49

0 437aa, >BBOV_III006470... *

1 437aa, >BBOV_III006510... at 98.86%

2 437aa, >BBOV_III006490... at 99.77%

>Cluster 50

0 437aa, >BBOV_III005800... *

1 437aa, >BBOV_III005620... at 94.05%

>Cluster 51

0 436aa, >BBOV_IV005950... *

>Cluster 52

0 433aa, >BBOV_III008960... *

>Cluster 53

0 432aa, >BBOV_III011590... *

>Cluster 54

0 431aa, >BBOV_IV010940... *

>Cluster 55

0 424aa, >BBOV_III011350... *

>Cluster 56

0 423aa, >BBOV_III007790... *

>Cluster 57

0 423aa, >BBOV_IV003350... *

>Cluster 58

0 421aa, >BBOV_IV008420... *

>Cluster 59

0 420aa, >BBOV_II002020... *

>Cluster 60

0 419aa, >BBOV_III001660... *

>Cluster 61

0 418aa, >BBOV_II000720... *

>Cluster 62

0 416aa, >BBOV_IV003320... *

>Cluster 63

0 416aa, >BBOV_III011320... *

>Cluster 64

0 414aa, >BBOV_III006100... *

>Cluster 65

0 411aa, >BBOV_III011330... *

>Cluster 66

0 404aa, >BBOV_I000820... *

>Cluster 67

0 395aa, >BBOV_IV009100... *

>Cluster 68

0 394aa, >BBOV_II003360... *

>Cluster 69

0 392aa, >BBOV_IV006420... *

>Cluster 70

0 373aa, >BBOV_IV001090... *

>Cluster 71

0 372aa, >BBOV_IV005390... *

>Cluster 72

0 371aa, >BBOV_I000970... *

>Cluster 73

0 361aa, >BBOV_I004780... *

>Cluster 74

0 360aa, >BBOV_IV008430... *

>Cluster 75

0 358aa, >BBOV_II002280... *

>Cluster 76

0 354aa, >BBOV_IV011680... *

>Cluster 77

0 354aa, >BBOV_IV004890... *

>Cluster 78

0 352aa, >BBOV_IV005440... *

>Cluster 79

0 351aa, >BBOV_IV004360... *

>Cluster 80

0 349aa, >BBOV_IV010280... *

>Cluster 81

0 346aa, >BBOV_IV008150... *

>Cluster 82

0 342aa, >BBOV_II002660... *

>Cluster 83

0 341aa, >BBOV_III003500... *

>Cluster 84

0 339aa, >BBOV_IV008060... *

>Cluster 85

0 339aa, >BBOV_II001640... *

>Cluster 86

0 326aa, >BBOV_II004060... *

>Cluster 87

0 323aa, >BBOV_III010990... *

>Cluster 88

0 323aa, >BBOV_III007330... *

>Cluster 89

0 312aa, >BBOV_IV005750... *

>Cluster 90

0 312aa, >BBOV_IV005870... *

>Cluster 91

0 308aa, >BBOV_IV010790... *

>Cluster 92

0 307aa, >BBOV_IV004210... *

>Cluster 93

0 300aa, >BBOV_IV003220... *

>Cluster 94

0 298aa, >BBOV_II002830... *

>Cluster 95

0 298aa, >BBOV_IV000670... *

>Cluster 96

0 297aa, >BBOV_IV011730... *

>Cluster 97

0 294aa, >BBOV_III006630... *

>Cluster 98

0 293aa, >BBOV_III007520... *

>Cluster 99

0 292aa, >BBOV_I003890... *

>Cluster 100

0 291aa, >BBOV_IV008620... *

>Cluster 101

0 285aa, >BBOV_III003290... *

>Cluster 102

0 282aa, >BBOV_III005720... *

>Cluster 103

0 282aa, >BBOV_III002860... *

>Cluster 104

0 281aa, >BBOV_I004040... *

>Cluster 105

0 280aa, >BBOV_III009100... *

>Cluster 106

0 277aa, >BBOV_III000800... *

>Cluster 107

0 276aa, >BBOV_IV001030... *

>Cluster 108

0 274aa, >BBOV_IV006500... *

>Cluster 109

0 274aa, >BBOV_IV006480... *

>Cluster 110

0 274aa, >BBOV_II002820... *

>Cluster 111

0 272aa, >BBOV_I001120... *

>Cluster 112

0 271aa, >BBOV_IV005650... *

>Cluster 113

0 268aa, >BBOV_III011700... *

>Cluster 114

0 267aa, >BBOV_I003500... *

>Cluster 115

0 265aa, >BBOV_I001680... *

>Cluster 116

0 265aa, >BBOV_I001670... *

>Cluster 117

0 262aa, >BBOV_II004430... *

>Cluster 118

0 259aa, >BBOV_IV002930... *

>Cluster 119

0 256aa, >BBOV_II007300... *

>Cluster 120

0 256aa, >BBOV_III002340... *

>Cluster 121

0 255aa, >BBOV_IV004340... *

>Cluster 122

0 253aa, >BBOV_III000390... *

>Cluster 123

0 252aa, >BBOV_II002580... *

>Cluster 124

0 250aa, >BBOV_IV000770... *

>Cluster 125

0 249aa, >BBOV_IV010000... *

>Cluster 126

0 248aa, >BBOV_I001660... *

>Cluster 127

0 248aa, >BBOV_IV010800... *

>Cluster 128

0 244aa, >BBOV_II002570... *

>Cluster 129

0 241aa, >BBOV_II006810... *

>Cluster 130

0 241aa, >BBOV_IV007930... *

>Cluster 131

0 240aa, >BBOV_IV004240... *

>Cluster 132

0 236aa, >BBOV_IV004850... *

>Cluster 133

0 236aa, >BBOV_IV009170... *

>Cluster 134

0 235aa, >BBOV_II003960... *

>Cluster 135

0 234aa, >BBOV_IV011310... *

>Cluster 136

0 233aa, >BBOV_IV003120... *

>Cluster 137

0 233aa, >BBOV_III000050... *

>Cluster 138

0 231aa, >BBOV_IV000780... *

>Cluster 139

0 228aa, >BBOV_IV008970... *

>Cluster 140

0 226aa, >BBOV_IV000420... *

>Cluster 141

0 221aa, >BBOV_IV009020... *

>Cluster 142

0 220aa, >BBOV_III009330... *

>Cluster 143

0 217aa, >BBOV_IV005000... *

>Cluster 144

0 217aa, >BBOV_I004270... *

>Cluster 145

0 217aa, >BBOV_IV004250... *

>Cluster 146

0 211aa, >BBOV_I002480... *

>Cluster 147

0 211aa, >BBOV_I001020... *

>Cluster 148

0 208aa, >BBOV_IV011670... *

>Cluster 149

0 204aa, >BBOV_IV008610... *

>Cluster 150

0 203aa, >BBOV_II001760... *

>Cluster 151

0 202aa, >BBOV_IV005180... *

>Cluster 152

0 196aa, >BBOV_IV012140... *

1 196aa, >BBOV_IV000040... at 91.84%

>Cluster 153

0 195aa, >BBOV_III010940... *

>Cluster 154

0 192aa, >BBOV_II000990... *

>Cluster 155

0 191aa, >BBOV_IV000760... *

>Cluster 156

0 189aa, >BBOV_I001370... *

>Cluster 157

0 188aa, >BBOV_I004140... *

>Cluster 158

0 188aa, >BBOV_IV009060... *

>Cluster 159

0 188aa, >BBOV_II006740... *

>Cluster 160

0 187aa, >BBOV_III011960... *

1 187aa, >BBOV_I005150... at 94.65%

>Cluster 161

0 182aa, >BBOV_I001170... *

>Cluster 162

0 182aa, >BBOV_I000840... *

>Cluster 163

0 178aa, >BBOV_IV001040... *

>Cluster 164

0 178aa, >BBOV_IV005120... *

>Cluster 165

0 173aa, >BBOV_I001070... *

>Cluster 166

0 172aa, >BBOV_IV011840... *

>Cluster 167

0 171aa, >BBOV_II005080... *

>Cluster 168

0 163aa, >BBOV_III010050... *

>Cluster 169

0 161aa, >BBOV_IV003250... *

>Cluster 170

0 159aa, >BBOV_III003930... *

>Cluster 171

0 159aa, >BBOV_I001130... *

>Cluster 172

0 156aa, >BBOV_IV000410... *

>Cluster 173

0 156aa, >BBOV_III000020... *

>Cluster 174

0 152aa, >BBOV_III002240... *

>Cluster 175

0 150aa, >BBOV_I004860... *

>Cluster 176

0 143aa, >BBOV_I002420... *

>Cluster 177

0 142aa, >BBOV_III000690... *

>Cluster 178

0 140aa, >BBOV_IV005880... *

>Cluster 179

0 140aa, >BBOV_II007780... *

>Cluster 180

0 140aa, >BBOV_IV000090... *

>Cluster 181

0 135aa, >BBOV_III001320... *

>Cluster 182

0 133aa, >BBOV_III009090... *

>Cluster 183

0 127aa, >BBOV_II002290... *

>Cluster 184

0 115aa, >BBOV_IV004940... *

>Cluster 185

0 108aa, >BBOV_III001690... *

>Cluster 186

0 108aa, >BBOV_III007710... *

>Cluster 187

0 108aa, >BBOV_IV008700... *

>Cluster 188

0 78aa, >BBOV_III002950... *

>Cluster 189

0 72aa, >BBOV_I001150... *

>Cluster 190

0 65aa, >BBOV_III001200... *

===

### Negative (non-exportome) proteins

Program: CD-HIT, V4.8.1 (+OpenMP), Mar 23 2021, 18:18:07

Command: ./cd-hit -i negatives_196.fa -o cdhit_negatives_196

Started: Tue Mar 23 18:47:27 2021

===

Output

----

total seq: 196

longest and shortest : 1821 and 51

Total letters: 81468

Sequences have been sorted

Approximated minimal memory consumption:

Sequence : 0M

Buffer : 1 X 10M = 10M

Table : 1 X 65M = 65M

Miscellaneous : 0M

Total : 76M

Table limit with the given memory limit:

Max number of representatives: 1163779

Max number of word counting entries: 90457824

comparing sequences from 0 to 196

196 finished 196 clusters

Approximated maximum memory consumption: 76M

writing new database

writing clustering information

program completed !

Total CPU time 0.06

===

>Cluster 0

0 1821aa, >BBOV_IV009200... *

>Cluster 1

0 1603aa, >BBOV_II003570... *

>Cluster 2

0 1437aa, >BBOV_III004820... *

>Cluster 3

0 1335aa, >BBOV_III002310... *

>Cluster 4

0 1211aa, >BBOV_III007730... *

>Cluster 5

0 1102aa, >BBOV_IV007980... *

>Cluster 6

0 1099aa, >BBOV_I005780... *

>Cluster 7

0 1040aa, >BBOV_III007250... *

>Cluster 8

0 1040aa, >BBOV_IV009720... *

>Cluster 9

0 1039aa, >BBOV_III003300... *

>Cluster 10

0 991aa, >BBOV_III005990... *

>Cluster 11

0 991aa, >BBOV_IV005090... *

>Cluster 12

0 991aa, >BBOV_I000250... *

>Cluster 13

0 988aa, >BBOV_II001010... *

>Cluster 14

0 959aa, >BBOV_III010710... *

>Cluster 15

0 957aa, >BBOV_IV011570... *

>Cluster 16

0 953aa, >BBOV_III008270... *

>Cluster 17

0 929aa, >BBOV_II004230... *

>Cluster 18

0 881aa, >BBOV_III009800... *

>Cluster 19

0 858aa, >BBOV_IV012020... *

>Cluster 20

0 846aa, >BBOV_IV005930... *

>Cluster 21

0 828aa, >BBOV_IV000880... *

>Cluster 22

0 815aa, >BBOV_II000760... *

>Cluster 23

0 804aa, >BBOV_IV008360... *

>Cluster 24

0 781aa, >BBOV_II003580... *

>Cluster 25

0 750aa, >BBOV_III004910... *

>Cluster 26

0 713aa, >BBOV_I004440... *

>Cluster 27

0 712aa, >BBOV_III004230... *

>Cluster 28

0 693aa, >BBOV_III003880... *

>Cluster 29

0 677aa, >BBOV_III009030... *

>Cluster 30

0 676aa, >BBOV_I001880... *

>Cluster 31

0 662aa, >BBOV_II007470... *

>Cluster 32

0 660aa, >BBOV_I004150... *

>Cluster 33

0 650aa, >BBOV_II006370... *

>Cluster 34

0 635aa, >BBOV_II002440... *

>Cluster 35

0 616aa, >BBOV_III009450... *

>Cluster 36

0 604aa, >BBOV_III007930... *

>Cluster 37

0 596aa, >BBOV_I002210... *

>Cluster 38

0 561aa, >BBOV_II003730... *

>Cluster 39

0 556aa, >BBOV_IV006790... *

>Cluster 40

0 548aa, >BBOV_II003080... *

>Cluster 41

0 546aa, >BBOV_II005220... *

>Cluster 42

0 542aa, >BBOV_IV004970... *

>Cluster 43

0 539aa, >BBOV_IV000260... *

>Cluster 44

0 538aa, >BBOV_II003390... *

>Cluster 45

0 537aa, >BBOV_III002510... *

>Cluster 46

0 535aa, >BBOV_IV007440... *

>Cluster 47

0 530aa, >BBOV_II003350... *

>Cluster 48

0 520aa, >BBOV_III009260... *

>Cluster 49

0 519aa, >BBOV_III004870... *

>Cluster 50

0 514aa, >BBOV_IV005830... *

>Cluster 51

0 508aa, >BBOV_IV005480... *

>Cluster 52

0 502aa, >BBOV_IV003870... *

>Cluster 53

0 495aa, >BBOV_IV006920... *

>Cluster 54

0 491aa, >BBOV_IV000400... *

>Cluster 55

0 485aa, >BBOV_III003170... *

>Cluster 56

0 485aa, >BBOV_IV010070... *

>Cluster 57

0 485aa, >BBOV_I003570... *

>Cluster 58

0 483aa, >BBOV_II006320... *

>Cluster 59

0 481aa, >BBOV_IV006780... *

>Cluster 60

0 476aa, >BBOV_II006920... *

>Cluster 61

0 474aa, >BBOV_III011560... *

>Cluster 62

0 468aa, >BBOV_III006620... *

>Cluster 63

0 462aa, >BBOV_III005110... *

>Cluster 64

0 453aa, >BBOV_IV004060... *

>Cluster 65

0 453aa, >BBOV_IV005840... *

>Cluster 66

0 445aa, >BBOV_II007650... *

>Cluster 67

0 444aa, >BBOV_III008310... *

>Cluster 68

0 443aa, >BBOV_IV008080... *

>Cluster 69

0 443aa, >BBOV_III011900... *

>Cluster 70

0 436aa, >BBOV_III007170... *

>Cluster 71

0 425aa, >BBOV_IV009940... *

>Cluster 72

0 423aa, >BBOV_II001020... *

>Cluster 73

0 423aa, >BBOV_IV001640... *

>Cluster 74

0 422aa, >BBOV_IV011970... *

>Cluster 75

0 418aa, >BBOV_III003080... *

>Cluster 76

0 416aa, >BBOV_IV005040... *

>Cluster 77

0 413aa, >BBOV_III004540... *

>Cluster 78

0 412aa, >BBOV_II004560... *

>Cluster 79

0 411aa, >BBOV_III008640... *

>Cluster 80

0 409aa, >BBOV_III006110... *

>Cluster 81

0 406aa, >BBOV_I002450... *

>Cluster 82

0 402aa, >BBOV_III010250... *

>Cluster 83

0 399aa, >BBOV_II004930... *

>Cluster 84

0 395aa, >BBOV_II007380... *

>Cluster 85

0	395aa, >BBOV_III010470... *

>Cluster 86

0	390aa, >BBOV_I002930... *

>Cluster 87

0	385aa, >BBOV_IV005540... *

>Cluster 88

0	379aa, >BBOV_IV003010... *

>Cluster 89

0	377aa, >BBOV_III011860... *

>Cluster 90

0	374aa, >BBOV_III006320... *

>Cluster 91

0	369aa, >BBOV_IV009590... *

>Cluster 92

0	364aa, >BBOV_IV001810... *

>Cluster 93

0	349aa, >BBOV_IV003080... *

>Cluster 94

0	348aa, >BBOV_IV008190... *

>Cluster 95

0	341aa, >BBOV_IV003860... *

>Cluster 96

0	339aa, >BBOV_II007560... *

>Cluster 97

0	336aa, >BBOV_I002490... *

>Cluster 98

0	329aa, >BBOV_I001610... *

>Cluster 99

0	329aa, >BBOV_II005400... *

>Cluster 100

0 323aa, >BBOV_III001060... *

>Cluster 101

0 321aa, >BBOV_III007940... *

>Cluster 102

0 320aa, >BBOV_I002820... *

>Cluster 103

0 314aa, >BBOV_III003950... *

>Cluster 104

0 312aa, >BBOV_IV004540... *

>Cluster 105

0 311aa, >BBOV_III004860... *

>Cluster 106

0 309aa, >BBOV_IV010530... *

>Cluster 107

0 308aa, >BBOV_III001540... *

>Cluster 108

0 306aa, >BBOV_III004840... *

>Cluster 109

0 302aa, >BBOV_I002910... *

>Cluster 110

0 301aa, >BBOV_II005860... *

>Cluster 111

0 297aa, >BBOV_II004540... *

>Cluster 112

0 293aa, >BBOV_I000910... *

>Cluster 113

0 292aa, >BBOV_IV000650... *

>Cluster 114

0 288aa, >BBOV_IV001650... *

>Cluster 115

0 281aa, >BBOV_IV001860... *

>Cluster 116

0 280aa, >BBOV_IV004160... *

>Cluster 117

0 276aa, >BBOV_IV003070... *

>Cluster 118

0 273aa, >BBOV_III003610... *

>Cluster 119

0 271aa, >BBOV_III005540... *

>Cluster 120

0 262aa, >BBOV_III002570... *

>Cluster 121

0 262aa, >BBOV_III010600... *

>Cluster 122

0 260aa, >BBOV_III004090... *

>Cluster 123

0 256aa, >BBOV_I000430... *

>Cluster 124

0 248aa, >BBOV_III007860... *

>Cluster 125

0 248aa, >BBOV_III009910... *

>Cluster 126

0 246aa, >BBOV_I004630... *

>Cluster 127

0 243aa, >BBOV_I004840... *

>Cluster 128

0 242aa, >BBOV_II002500... *

>Cluster 129

0 242aa, >BBOV_II000620... *

>Cluster 130

0 239aa, >BBOV_III008280... *

>Cluster 131

0 238aa, >BBOV_IV010500... *

>Cluster 132

0 236aa, >BBOV_IV003270... *

>Cluster 133

0 234aa, >BBOV_IV011510... *

>Cluster 134

0 230aa, >BBOV_III004660... *

>Cluster 135

0 225aa, >BBOV_II001250... *

>Cluster 136

0 225aa, >BBOV_II004070... *

>Cluster 137

0 223aa, >BBOV_IV000600... *

>Cluster 138

0 221aa, >BBOV_V000090... *

>Cluster 139

0 217aa, >BBOV_IV010550... *

>Cluster 140

0 214aa, >BBOV_V000540... *

>Cluster 141

0 213aa, >BBOV_II005810... *

>Cluster 142

0 213aa, >BBOV_III005750... *

>Cluster 143

0 212aa, >BBOV_I001770... *

>Cluster 144

0 211aa, >BBOV_III001740... *

>Cluster 145

0 207aa, >BBOV_III004170... *

>Cluster 146

0 207aa, >BBOV_II006260... *

>Cluster 147

0 201aa, >BBOV_III007640... *

>Cluster 148

0 196aa, >BBOV_IV000530... *

>Cluster 149

0 195aa, >BBOV_I005030... *

>Cluster 150

0 193aa, >BBOV_I000240... *

>Cluster 151

0 190aa, >BBOV_I002860... *

>Cluster 152

0 188aa, >BBOV_II004080... *

>Cluster 153

0 187aa, >BBOV_III007190... *

>Cluster 154

0 180aa, >BBOV_IV004220... *

>Cluster 155

0 176aa, >BBOV_IV002410... *

>Cluster 156

0 175aa, >BBOV_III006650... *

>Cluster 157

0 175aa, >BBOV_II005040... *

>Cluster 158

0 173aa, >BBOV_I003190... *

>Cluster 159

0 169aa, >BBOV_III010670... *

>Cluster 160

0 167aa, >BBOV_IV000800... *

>Cluster 161

0 164aa, >BBOV_III009860... *

>Cluster 162

0 163aa, >BBOV_I001760... *

>Cluster 163

0 162aa, >BBOV_I005050... *

>Cluster 164

0 159aa, >BBOV_IV008910... *

>Cluster 165

0 152aa, >BBOV_IV004930... *

>Cluster 166

0 151aa, >BBOV_IV006660... *

>Cluster 167

0 149aa, >BBOV_I004080... *

>Cluster 168

0 146aa, >BBOV_III010680... *

>Cluster 169

0 145aa, >BBOV_IV005100... *

>Cluster 170

0 145aa, >BBOV_I001340... *

>Cluster 171

0 141aa, >BBOV_II005000... *

>Cluster 172

0 135aa, >BBOV_II003270... *

>Cluster 173

0 134aa, >BBOV_III006250... *

>Cluster 174

0 132aa, >BBOV_III001350... *

>Cluster 175

0 129aa, >BBOV_V000300... *

>Cluster 176

0 128aa, >BBOV_IV002190... *

>Cluster 177

0 127aa, >BBOV_IV002860... *

>Cluster 178

0 125aa, >BBOV_IV007200... *

>Cluster 179

0 120aa, >BBOV_II007490... *

>Cluster 180

0 118aa, >BBOV_III007130... *

>Cluster 181

0 113aa, >BBOV_III009270... *

>Cluster 182

0 112aa, >BBOV_IV004410... *

>Cluster 183

0 111aa, >BBOV_III007490... *

>Cluster 184

0 110aa, >BBOV_I004740... *

>Cluster 185

0 110aa, >BBOV_I002260... *

>Cluster 186

0 94aa, >BBOV_IV002350... *

>Cluster 187

0 90aa, >BBOV_IV003900... *

>Cluster 188

0 87aa, >BBOV_IV009630... *

>Cluster 189

0 77aa, >BBOV_II003780... *

>Cluster 190

0 76aa, >BBOV_III002410... *

>Cluster 191

0 75aa, >BBOV_I002150... *

>Cluster 192

0 73aa, >BBOV_I002430... *

>Cluster 193

0 70aa, >BBOV_III000120... *

>Cluster 194

0 59aa, >BBOV_I001590... *

>Cluster 195

0 51aa, >BBOV_II004750... *
