# Supplementary material for: Predicting Protein Therapeutic Candidates for Bovine Babesiosis Using Secondary Structure Properties and Machine Learning
Source: Front Genet. 2021 Jul 23;12:716132. doi: 10.3389/fgene.2021.716132 (PMC8343536; doi:10.3389/fgene.2021.716132)
Supplement: Supplementary file 3 [file Image_1.PDF]

Supplementary Figure S1

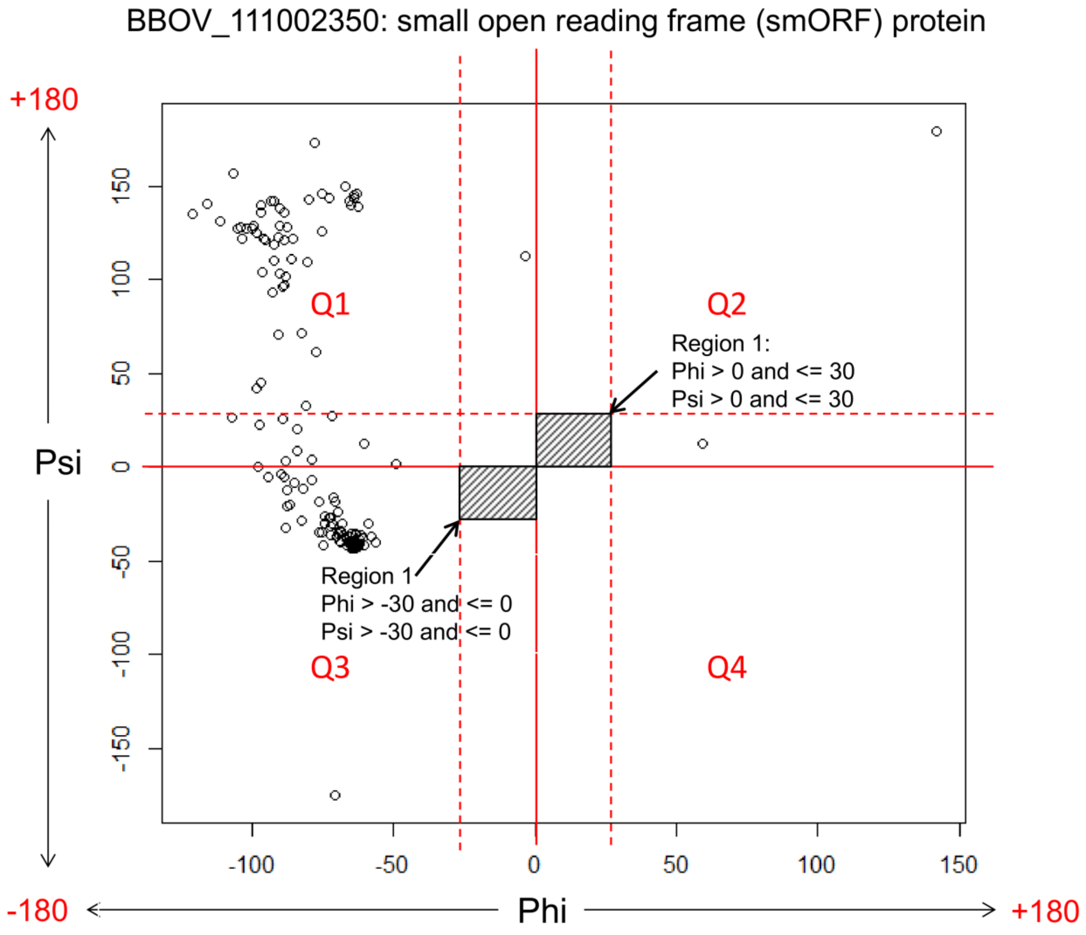

This is a plot of a protein's backbone torsion (dihedral) angles ϕ (Phi) and ψ (Psi). In this example, the protein BBOV_III002350 is a small open reading frame (smORF) protein. It is expected to be an exportome member because smORF proteins are known to have an association with the erythrocyte membrane. Phi and Psi are values between -180 and +180. The small clear circles represent the 'Psi vs Phi' angles derived from all 142 residues of BBOV_III002350. The plot is divided into four quadrants (Q1, Q2, Q3, and Q4) and then each quadrant is subdivided into regions. In this illustration, one squared region has a dimension of 30 e.g. Region 1 in Q2 is defined by Phi angles > 0 and <= 30 *and* Psi angles > 0 and <= 30. There are 36 regions per quadrant and therefore 144 in total for the entire plot. Each region represents a feature for machine learning input, whereby the feature value is the number of residues within the region.
