# Supplementary material for: Predicting Protein Therapeutic Candidates for Bovine Babesiosis Using Secondary Structure Properties and Machine Learning
Source: Front Genet. 2021 Jul 23;12:716132. doi: 10.3389/fgene.2021.716132 (PMC8343536; doi:10.3389/fgene.2021.716132)
Supplement: Supplementary file 8 [file Table_5.PDF]

Supplementary Table S5

Machine learning performance measures for predicting the presence of transmembrane domains

Performance measures (%)	adaBoost	RF	Ensemble
Accuracy	72.91	**76.25**	73.65
Error Rate	27.09	**23.75**	26.35
Sensitivity	71.06	**72.73**	71.61
False Positive Rate	25.23	**20.22**	24.30
Specificity	74.77	**79.78**	75.70
Positive Predictive Value	73.80	**78.24**	74.66
Negative Predictive Value	72.09	**74.52**	72.73

Values in bold indicate best performances.

adaBoost = adaptive boosting; RF = Random Forest ; Ensemble = final classifications derived from the average of adaBoost and RF classification probabilities.
