# Supplementary material for: Predicting Protein Therapeutic Candidates for Bovine Babesiosis Using Secondary Structure Properties and Machine Learning
Source: Front Genet. 2021 Jul 23;12:716132. doi: 10.3389/fgene.2021.716132 (PMC8343536; doi:10.3389/fgene.2021.716132)
Supplement: Supplementary file 9 [file Table_6.PDF]

Supplementary Table S6

Percentage of prediction misclassifications and the accuracy per secondary structure prediction method for *Babesia bovis* T2Bo test data when using only Spider3 data

	[b]Spider only			[C]Multiple predictors		
**Prediction method**	**Positives (%)**	**Negatives (%)**	**Accuracy (%)**	**Positives (%)**	**Negatives (%)**	**Accuracy (%)**
3 classes	18.8	16.3	82.5	10.0	11.3	89.4
8 classes	17.5	16.3	83.1	8.8	15.0	86.3
Phi and psi angles	21.3	12.5	83.1	13.8	11.3	87.5
solvent-Accessible Surface Area	3.8	10.0	93.1	3.8	10.0	93.1
Half-Sphere Exposure – upper sphere	3.8	12.5	91.9	2.5	12.5	92.5
**[a]Average**	**2.5**	**5.0**	**96.2**	**3.8**	**5.0**	**95.6**

Test data = 80 *Babesia bovis* T2Bo proteins that meet the Gohil rule-based selection but not included in training data (positives), and 80 *Babesia bovis* T2Bo proteins that fail the Gohil rule-based selection criteria but not included in training data (negatives).

[a]Average = average exportome probabilities for all five prediction methods (3 classes, 8 classes, phi and psi angles, solvent-Accessible Surface Area (ASA), and Half-Sphere Exposure (HSE) – upper sphere).

[b]Spider only = Only the Spider3 predictor was used to generate the required secondary structure predication inputs (3 and 8 classes, psi and phi angles, solvent-accessible surface area, and half-sphere exposure – upper sphere)

[b]Multiple predictors = The source of the 3 and 8 classes was obtained from a consensus of nine and seven predictors respectively; psi and phi angle predictions obtained from a consensus of three predictors (SPOT-1D, NetsurfP, and MUFold); and ASA and HSE-upper predictions from Spider3

Positives (%) = percentage of positive classifications per method different to that expected; Negatives (%) = percentage of negative classifications per method different to that expected.
