# Supplementary material for: Predicting Protein Therapeutic Candidates for Bovine Babesiosis Using Secondary Structure Properties and Machine Learning
Source: Front Genet. 2021 Jul 23;12:716132. doi: 10.3389/fgene.2021.716132 (PMC8343536; doi:10.3389/fgene.2021.716132)
Supplement: Supplementary file 10 [file Table_7.PDF]

Supplementary Table S7

Breakdown of exportome membership predictions for *Babesia bovis* T2Bo when using Spider3 input data.

Preface: exportome membership probabilities were predicted for 3154 *Babesia bovis* T2Bo proteins using the five secondary structure predictions methods with Spider3 input data i.e. 3706 *Babesia bovis* T2Bo proteins - 392 training proteins – 160 test proteins = 3154

With respect to the 3154 proteins and their predicted membership probability: 550 >= 0.5, 166 >= 0.7, 88 => 0.8, 29 >= 0.9.

Of the 166 proteins with exportome membership probability >= 0.7, 115 had a consensus of 5 (i.e. all 5 prediction methods had exportome membership probabilities >= 0.5), 49 had a consensus of 4, and 2 had a consensus of 3.

The following is a breakdown of the 166 proteins (minus the two consensus 3 proteins) with reference to the presence of predicted signal peptides and transmembrane domains:

**Description**[a]	**All**[b]	**Spider3**[c]	**TM**[d]
Signal peptide	368	53	27
Signal peptide and at least 1 transmembrane domain	102	47	23
Signal peptide and only 1 transmembrane domain	58	20	3
Signal peptide and at least 1 transmembrane domain located in first 60 amino acids	69	4	0
Signal peptide and 1 transmembrane domain located in first 60 amino acids	33	4	0
Signal peptide and no transmembrane domain	266	7	4
At least 1 transmembrane domain irrespective of signal peptide	677	74	37
no signal peptide and at least 1 transmembrane domain	575	28	14
No signal peptide and no transmembrane domains	2763	83	18
Potential vaccine candidates[e]		101	0

[a]Presence of signal peptide predicted by SignalP 5.0 with threshold > 0.5, and the number of transmembrane domains contained in a protein predicted by TMHMM 2.0;[b]protein count out of a possible 3706 *Babesia bovis* T2Bo proteins currently available; [c]protein count out of a possible 166 *Babesia bovis* T2Bo proteins with an exportome membership probability >= 0.7; [d]Number of proteins containing a transmembrane (TM) domain as predicted by machine learning with Spider3 derived backbone torsion angles ϕ (phi) and ψ (psi) as input; [e]101 out of the 166 proteins were selected as possible vaccine candidates i.e. exportome membership probability >= 0.7 and 0 predicted TMs.
