# Supplementary material for: Predicting Protein Therapeutic Candidates for Bovine Babesiosis Using Secondary Structure Properties and Machine Learning
Source: Front Genet. 2021 Jul 23;12:716132. doi: 10.3389/fgene.2021.716132 (PMC8343536; doi:10.3389/fgene.2021.716132)
Supplement: Supplementary file 12 [file Table_9.PDF]

Supplementary Table S9

Breakdown of exportome membership predictions for test species

Test species	Total	Rule-based[a]	ML-SS 'vs' Rule-based[b]	ML-SS from Total[c]
Babesia bigemina BOND	5077	277	255 (92.1%)	441 (327)
Babesia canis BcH-CHIPZ	3467	133	117 (87.9%)	214 (156)
Plasmodium falciparum 3D7	5460	264	259 (98.1%)	718 (343)
Plasmodium falciparum 3D7 STEVOR and RIFIN	RIFIN 33<br>STEVOR 3	RIFIN 0<br>STEVOR 1	RIFIN 33<br>STEVOR 3	RIFIN 128 (0)[d]<br>STEVOR 13 (0)[e]
Toxoplasma gondii ME49	8322	318	275 (86.5%)	620 (523)
Toxoplasma gondii ME49 GRA and ROP	GRA 18<br>ROP 19	GRA 7<br>ROP 5	GRA 9<br>ROP 10	GRA 7 (5)[f]<br>ROP 10 (9)[g]

RIFIN = *Plasmodium* protein families, repetitive interspersed family, STEVOR = subtelomeric variable open reading frame family, GRA = dense granule, and ROP = rhoptry protein.

[a]Number of proteins out of the Total that meet the Gohil rule-based selection criteria, [b] Number of proteins (percentage in brackets) out of the Rule-based total that have an average exportome membership probability > 0.5 as predicted by the five machine learning secondary structure (ML-SS) predictions methods (3 and 8 classes, psi and phi angles, ASA, and HSE-upper) , [c]Number of proteins out of the Total that have a predicted average exportome membership probability > 0.7 (enclosed brackets contains this number minus the number of proteins with a transmembrane domain warning > 0.5); [d]128 out of 157 proteins named 'rifin' have a predicted average exportome membership probability > 0.7 (155 > 0.5). All RIFIN proteins contain at least one transmembrane domain as predicted by TMHMM hence the 0 in brackets, and 52 have no signal peptide as predicted by SignalP; [e]13 out of 33 proteins named 'stevor' or 'stevor-like' have a predicted average exportome membership probability > 0.7 (30 > 0.5). All STEVOR proteins contain at least one transmembrane domain as predicted by TMHMM, and 11 have no signal peptide as predicted by SignalP; [f]7 out of 18 proteins with 'dense granular protein' in the name have a predicted average exportome membership probability > 0.7 (9 > 0.5); [g]10 out of 19 proteins with 'rhoptry protein' in the name have a predicted average exportome membership probability > 0.7 (10 > 0.5).
