# Supplementary material for: Predicting Protein Therapeutic Candidates for Bovine Babesiosis Using Secondary Structure Properties and Machine Learning
Source: Front Genet. 2021 Jul 23;12:716132. doi: 10.3389/fgene.2021.716132 (PMC8343536; doi:10.3389/fgene.2021.716132)
Supplement: Supplementary file 13 [file Table_10.PDF]

Supplementary Table S10

Predicted candidates for test species using machine learning and secondary structure properties

Test species	Total	>0.5	>0.7	>0.7 - Warn	No SP	TMs	Expect
Babesia bovis T2Bo	3154[a]	549	166	101	75	19	81.2%
Babesia bigemina BOND	5077	1174	441[b]	327[c]	106	41	87.5%
Babesia canis BcH-CHIPZ	3467	484	214[d]	155[e]	74	18	88.4%
Plasmodium falciparum 3D7	5460	1640	718[f]	372[g]	153	74	80.1%
Toxoplasma gondii ME49	8322	1777	620	523	226	66	87.4%

[a]*Babesia bovis* T2Bo has 3706 available proteins. Training and test proteins were excluded to give a total of 3154; [b]Includes HSP70, HSP90, SBP3, SBP4, MAC/perforin, [c] Includes all 'b' proteins; [d]Includes HSP90 and MAC/perforin; [e] Includes all 'd' proteins; [f]Includes HSP70, HSP90, HSP110, HSP20-like chaperone, and *Plasmodium* exported proteins containing helical interspersed subtelomeric (PHIST) or HYP domains; [g] Includes all 'f' proteins except 5 of the 8 PHIST and HYP proteins.

>0.5 or >0.7 = threshold applied to the average exportome membership probability as predicted by the five secondary prediction methods (3 and 8 classes, psi and phi angles, ASA, and HSE-upper); - Warn = proteins predicted with a transmembrane (TM) domain presence indicator > 0.5 were filtered from the >0.7 pool of proteins; No SP = number of >0.7 filtered proteins with no signal peptide as predicted by SignalP; TMs = number of >0.7 filtered proteins with at least one transmembrane domain as predicted by TMHMM; Expect = percentage of >0.7 filtered proteins expected to be correct based on a manual assessment when considering the number of TMs.

SBP = spherical body proteins, HSP = heat shock proteins; MAC/perforin = membrane attack complex (MAC)/perforin or DnaJ domain containing protein.
