# Supplementary material for: Predicting Protein Therapeutic Candidates for Bovine Babesiosis Using Secondary Structure Properties and Machine Learning
Source: Front Genet. 2021 Jul 23;12:716132. doi: 10.3389/fgene.2021.716132 (PMC8343536; doi:10.3389/fgene.2021.716132)
Supplement: Supplementary file 14 [file Table_11.PDF]

Supplementary Table S11

Breakdown of the number of *Babesia bovis* T2Bo proteins containing signal peptides and/or transmembrane domains

**Description**[a]	**Count**[b]
Signal peptide	368
Signal peptide and at least 1 transmembrane domain	102
Signal peptide and only 1 transmembrane domain	58
Signal peptide and at least 1 transmembrane domain located in first 60 amino acids	69
Signal peptide and 1 transmembrane domain located in first 60 amino acids	33
Signal peptide and no transmembrane domain	266
At least 1 transmembrane domain irrespective of signal peptide	677
no signal peptide and at least 1 transmembrane domain	575
No signal peptide and no transmembrane domains	2763

[a]Presence of signal peptide predicted by SignalP 5.0 with threshold > 0.5, and number of transmembrane domains contained in a protein predicted by TMHMM 2.0;[b]Protein count out of a possible 3706 *Babesia bovis* T2Bo proteins currently available (protein lengths 59 – 2953).
